# A multi-resource data integration approach: identification of candidate genes regulating cell proliferation during neocortical development

**DOI:** 10.3389/fnins.2014.00257

**Published:** 2014-08-21

**Authors:** Cynthia M. Vied, Florian Freudenberg, Yuting Wang, Alexandre A. S. F. Raposo, David Feng, Richard S. Nowakowski

**Affiliations:** ^1^Department of Biomedical Sciences, College of Medicine, Florida State UniversityTallahassee, FL, USA; ^2^Department of Psychiatry, Psychosomatics and Psychotherapy, University Hospital of FrankfurtFrankfurt, Germany; ^3^NUS Graduate School for Integrative Sciences and Engineering, National University of SingaporeSingapore, Singapore; ^4^Molecular Neurobiology, Instituto Gulbenkian de CiênciaOeiras, Portugal; ^5^Allen Institute for Brain ScienceSeattle, WA, USA

**Keywords:** the allen institute for brain science, neocortex development, gene expression, ventricular zone (VZ), subventricular zone (SVZ), GeneMANIA

## Abstract

Neurons of the mammalian neocortex are produced by proliferating cells located in the ventricular zone (VZ) lining the lateral ventricles. This is a complex and sequential process, requiring precise control of cell cycle progression, fate commitment and differentiation. We have analyzed publicly available databases from mouse and human to identify candidate genes that are potentially involved in regulating early neocortical development and neurogenesis. We used a mouse *in situ* hybridization dataset (The Allen Institute for Brain Science) to identify 13 genes (*Cdon*, *Celsr1*, *Dbi*, *E2f5*, *Eomes*, *Hmgn2*, *Neurog2*, *Notch1*, *Pcnt*, *Sox3*, *Ssrp1*, *Tead2*, *Tgif2*) with high correlation of expression in the proliferating cells of the VZ of the neocortex at early stages of development (E15.5). We generated a similar human brain network using microarray and RNA-seq data (BrainSpan Atlas) and identified 407 genes with high expression in the developing human VZ and subventricular zone (SVZ) at 8–9 post-conception weeks. Seven of the human genes were also present in the mouse VZ network. The human and mouse networks were extended using available genetic and proteomic datasets through GeneMANIA. A gene ontology search of the mouse and human networks indicated that many of the genes are involved in the cell cycle, DNA replication, mitosis and transcriptional regulation. The reported involvement of *Cdon*, *Celsr1*, *Dbi, Eomes*, *Neurog2*, *Notch1*, *Pcnt*, *Sox3*, *Tead2*, and *Tgif2* in neural development or diseases resulting from the disruption of neurogenesis validates these candidate genes. Taken together, our knowledge-based discovery method has validated the involvement of many genes already known to be involved in neocortical development and extended the potential number of genes by 100's, many of which are involved in functions related to cell proliferation but others of which are potential candidates for involvement in the regulation of neocortical development.

## Introduction

The neurons of the mammalian neocortex originate from neural progenitor cells (NPCs, either neuroepithelial cells or apical radial glia derived from the former) (Hartfuss et al., 2001; Kriegstein and Götz, 2003; Götz and Huttner, 2005). Initially, NPCs undergo symmetric proliferative divisions in the ventricular zone (VZ) of the dorsal telencephalon, amplifying their population. Cortical neurogenesis begins when these cells switch to differentiative asymmetric division, resulting in one daughter cell remaining an NPC and the other emerging as either a committed progenitor lineage or a postmitotic neuron (Götz and Huttner, 2005; Huttner and Kosodo, 2005). The intermediate progenitors move to the subventricular zone (SVZ), where they form an additional neurogenic layer that supplies projection neurons to all layers of the cerebral cortex (Takahashi et al., 1994, 1995, 1999; Haubensak et al., 2004; Kowalczyk et al., 2009). In addition to their role as progenitor cells, the bipolar apical radial glia at the VZ extend pial-directed processes which guide the migration of both intermediate progenitors and newly generated neurons (Ayala et al., 2007).

The VZ plays a central role in a complex and sequential process, requiring precise control of cell cycle progression, fate commitment, differentiation, and migration. Symmetric or asymmetric localization of subcellular components (Morin and Bellaïche, 2011), the Par complex (Wirtz-Peitz et al., 2008), signaling pathways including Notch (De la Pompa et al., 1997; Caviness et al., 2009), and the intercellular FGF and Wnt signaling pathways all interact with each other to regulate both proliferation and differentiation in the VZ of the neocortex (Johansson et al., 2010).

The advent of whole genome technologies, particularly microarray and RNA-seq methods, has produced a wealth of information on the mammalian brain which has increasingly been deposited in publicly available databases. Consequently, we are now able to move from individual interactions to the identification of thousands of genes that are involved in biological processes. In this study, we used a knowledge-based discovery approach, analyzing publicly available databases from both mouse and human to identify candidate genes that are potentially involved in regulating early neocortical development and neurogenesis. Using this approach we have generated a network of 13 genes from mouse that are expressed in the VZ during early neocortical development. We also found over 400 candidate genes from human that are expressed in the VZ and SVZ at a comparable developmental time. As expected, many of these genes are known to be involved in the cell cycle, transcription and DNA replication. In addition, we used GeneMANIA and literature mining to generate extended mouse and human networks and to determine that at least two genes from the mouse network are potential novel regulators of neocortical development. This implies that some of the 400 human genes we identified could be novel regulators of early cortical development and could be the basis for future experimental studies.

## Materials and methods

### Differential gene expression using the allen developing mouse brain atlas

To identify genes with high differential expression in the VZ of the developing mouse neocortex, we used the Allen Developing Mouse Brain Atlas (Thompson et al., 2014) (^©^ 2013 Allen Institute for Brain Science. Allen Developing Mouse Brain Atlas. Available from: http://developingmouse.brain-map.org/). This atlas provides spatial expression patterns from *in situ* hybridization (ISH) images for 2104 genes related to brain development at multiple stages ranging between embryonic day 11.5 (E11.5) and postnatal day 56 (P56) of C57BL/6J mice. Specifically, we made use of the Allen Brain Atlas application programming interface (API) (http://www.brain-map.org/api/index.html) to identify genes that are: (1) expressed in the telencephalon at E15.5, and (2) highly expressed in the VZ. The API's correlation search service yielded correlation scores for these genes. The most tightly correlated genes (*r* > 0.7) were used to create a Neocortex VZ Network. We analyzed expression of these genes in a qualitative manner by downloading high resolution ISH images for these genes from the Allen Developing Mouse Brain Atlas (http://help.brain-map.org/download/attachments/4325389/DevMouse_Overview.pdf).

In addition, analogous to the VZ network, we analyzed the Allen Mouse Brain Atlas for genes with specific expression in the neocortex outside of the VZ (Non-VZ Network; Figure S1). We did not further characterize the genes in the non-VZ network.

### Identification of differentially expressed genes using the BrainSpan atlas

We identified genes differentially expressed in the VZ and SVZ of the human brain early in development using the BrainSpan Atlas of the Developing Human Brain (Miller et al., 2014; ^©^ 2014 Allen Institute for Brain Science. BrainSpan Atlas of the Developing Human Brain. Available from: http://brainspan.org/). Specifically, we used the developmental transcriptome dataset, which is comprised of RNA sequencing and exon microarray data of ~52,400 genes from brains at different developmental stages [8 post-conceptual weeks (pcw) up to 39 years of age] to identify genes differentially expressed at 8–9 pcw vs. all later stages. We used the prenatal laser microdissection (LMD) microarray dataset (comprised of microarray profiles from ~58,700 probes taken from ~300 different brain structures between 15 and 21 pcw) to find genes differentially expressed in the VZ and SVZ relative to the rest of the brain. For both datasets (developmental transcriptome and prenatal LMD microarray) we used the BrainSpan web interface to download fold-change and *p*-values for differentially expressed genes. Only those genes with at least six-fold higher expression (developmental transcriptome dataset: six-fold higher expression at 8–9 pcw vs. all later stages; LMD microarray dataset: six-fold higher expression in the VZ and SVZ compared to the rest of the brain) were used. Subsequently we corrected *p*-values using the Holm-Bonferroni method and set the significant *p*-value at 1 × 10^−9^. We then compared the resulting lists for overlapping genes and used these genes for further analysis.

### GeneMANIA association network

GeneMANIA (http://genemania.org/) (Mostafavi et al., 2008) was used to find genes that are related to the 13 mouse VZ network genes (**Figure 2A**) and the 407 human network genes (**Figure 5A**). GeneMANIA uses functional association data including protein and genetic interactions, pathways, co-expression, co-localization, protein domain similarity and predicted interactions (based on organisms other than the one searched). GeneMANIA recommends using gene lists with no more than 100 genes for a search. Therefore, we used the top 100 genes from the human network plus the 7 mouse genes that overlapped between the mouse and human lists. All 13 mouse genes were searched through GeneMANIA. An additional 50 genes were shown in each network that are associated with the original 13 VZ network genes or the 407 human network genes. The parameters used for the search, genes queried, associated gene lists, references used to generate the associations and all other outputs from GeneMANIA can be found in Tables S1,S4 for the mouse and human searches, respectively. The database version for these searches was June 1, 2014 and default settings were used for the appropriate organism for each list.

### Gene ontology and pathway enrichment analysis

WebGestalt, (http://bioinfo.vanderbilt.edu/webgestalt/; Zhang et al., 2005; Wang et al., 2013) and GOrilla (Eden et al., 2007, 2009), two web-based enrichment analysis platforms, were used to determine pathway and gene ontology (GO) enrichment. For pathway analysis in WebGestalt, a list of 407 genes correlated from the LMD microarray dataset and the developmental transcriptome was provided and the hsapiens_genome was used as the reference set to obtain significantly enriched [false discovery rate (FDR) adjusted *p*-value <0.05] pathways. Through WebGestalt we chose the Pathway Commons (Cerami et al., 2011) enrichment analysis. The statistical test used was hypergeometric and the Benjamini-Hochberg FDR method was used for multiple testing adjustment. GOrilla (http://cbl-gorilla.cs.technion.ac.il/) was used to identify GO terms that are significantly overrepresented in the list of 63 mouse extended network genes and 407 human SVZ and VZ genes. The mouse search was relative to a background set of the mouse genome and the human search was relative to approximately 18,000 human genes that could be associated with any GO term. GOrilla results were visualized as “TreeMaps” generated in REViGO (http://revigo.irb.hr/; Supek et al., 2011).

### Literature mining

To validate the potential interaction among the 13 VZ network genes, we performed a literature mining approach on published studies to generate a hypothetical functional gene network (**Figure 6**). A PubMed search was carried out using the keywords: (1) 13 gene symbols respectively; (2) combination of any two of the 13 gene symbols (e.g., *Celsr1*, *Dbi*). Specifically, “Cdo” and “Cdon” were used for *Cdon* studies, as “*Cdo*” was used as the gene symbol previously. Advanced search by “[Title/Abstract]” was performed for *Notch1* because the number of articles was 3269 (indicated with “*”). Numbers of articles retrieved are listed in Table S6A. Titles, abstracts and “Materials and Methods” were examined to exclude non-mammalian or non-neuronal studies. Twenty-eight genes were identified that have interactions with the 13 candidates (Table S6B). The combination of any two of those genes were used as keywords in PubMed search (Table S6B). Advanced search by “[Title/Abstract]” was also used in some cases to control the number of studies (indicated with “^*^”). Non-mammalian or non-neuronal studies were excluded by scrutinizing titles, abstracts and “Materials and Methods.” The hypothetical functional gene network contains the 13 candidates and a subset of the 28 genes that interacted with more than one gene found through the literature mining approach (Pani et al., 2002; Scardigli, 2003; Li et al., 2004, 2012a,b; Schuurmans et al., 2004; Yang et al., 2004; Taranova et al., 2006; Allen et al., 2007; Taylor et al., 2007; Nakazaki et al., 2008; Sawada et al., 2008; Shimizu et al., 2008; Shimojo et al., 2008; Wen et al., 2008; Yu et al., 2008; Favaro et al., 2009; Fernandez et al., 2009; Henke et al., 2009; Ochiai et al., 2009; Aguirre et al., 2010; Hu et al., 2010; Kaltezioti et al., 2010; Qu et al., 2010, 2013; Chavali et al., 2011; Gee et al., 2011; Karalay et al., 2011; Sinor-Anderson and Lillien, 2011; Taniguchi et al., 2012; Xia et al., 2012; Zhao et al., 2012, 2014; Zhang et al., 2012a,b, 2013; Imamura and Greer, 2013; Marqués-Torrejón et al., 2013; Petrova et al., 2013; Misra et al., 2014). Note that we listed only one article for each interaction (selection priority (original research articles only): *in vivo* > *in vitro;* direct interaction > indirect interaction). However, for some correlations more than one article is accessible.

## Results

### Mouse neocortex ventricular zone network

**Figure 1:**
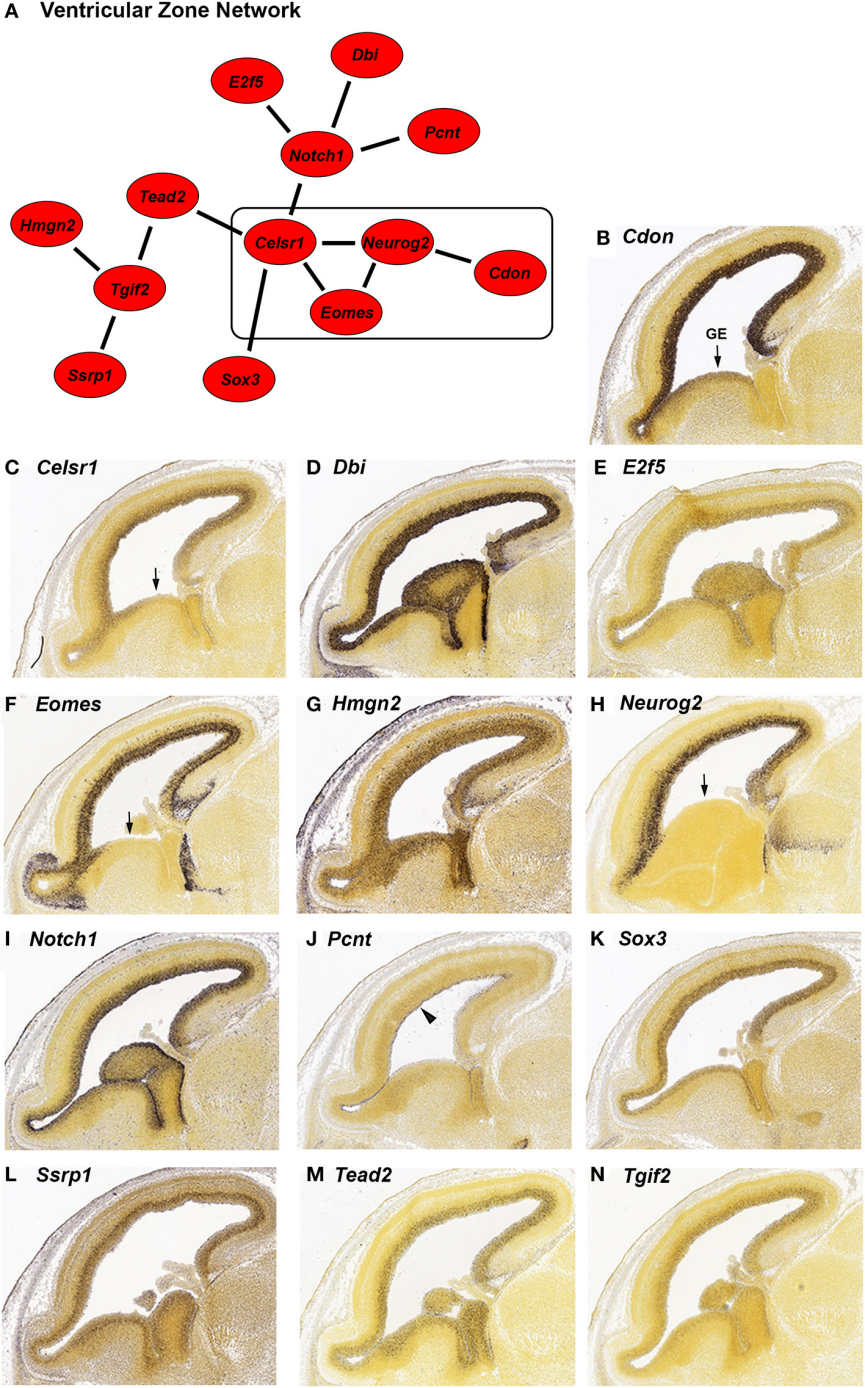
***In situ* hybridization (ISH) of genes highly expressed in the ventricular zone (VZ) during embryonic development from the Allen Developing Mouse Brain Atlas. (A)** Network of genes with high differential expression in the VZ of the mouse brain at age E15.5. The strength of correlation between the genes was at least an *r*-value of 0.7. The rectangle indicates genes with reduced expression in the ganglionic eminence (GE). **(B–N)** Individual ISH images of genes from the VZ network **(A)**. All genes show preferential expression in the proliferating cells of the VZ. *Pcnt* **(J)** is specifically expressed on the lateral wall of the lateral ventricle (indicated by the arrowhead). Four genes; *Cdon* **(B)**, *Celsr1* **(C)**, *Eomes* **(F)**, and *Neurog2* **(H)** have decreased expression in the GE (indicated by arrows).

To identify genes that are involved in neocortex development, we generated a network of genes that are differentially expressed in the VZ of the mouse embryonic neocortex (Figure 1A). We performed a correlation search for genes with similar spatial expression patterns using the API of the Allen Developing Mouse Brain Atlas from The Allen Institute for Brain Science (http://developingmouse.brain-map.org/). This atlas has ISH data for over 2100 developmentally important genes during seven stages of development beginning at E11.5 through P56. We specifically analyzed data from E15.5, which is during the peak of neurogenesis for neocortex development. We graphed the most tightly correlated genes that are differentially expressed in the VZ of the neocortex at that time point (i.e., E15.5) compared to all other time points and brain areas (Figure 1). It should be noted that less than 10% of the protein-coding genes are represented in the Allen Developing Mouse Brain Atlas and therefore more genes could be expected in this network if all mouse genes were included.

We identified 13 genes in this search; *Cdon*, *Celsr1*, *Dbi*, *E2f5*, *Eomes*, *Hmgn2*, *Neurog2*, *Notch1*, *Pcnt*, *Sox3*, *Ssrp1*, *Tead2*, and *Tgif2*. We verified the expression and expression pattern of the 13 genes in the neocortex at E15.5 using the ISH data from the Developing Mouse Brain Atlas (Figures 1B–N). All 13 genes are predominantly expressed in the VZ of the neocortex. Three of those genes, *Pcnt*, *Ssrp1*, and *Tead2* seem to be expressed in only a subset of the VZ cells. *Pcnt* (*pericentrin*) is expressed on the surface of the lateral ventricle (Figure 1J, arrowhead). This expression of *Pcnt* was observed previously (Miyoshi et al., 2006), however the functional consequences of this expression pattern are unknown. The same group reported PCNT protein localization at the base of primary cilia in E13 developing cerebral cortex, which is consistent with a role for *Pcnt* in microtubule organization (Miyoshi et al., 2006). The expression of *Ssrp1* is higher in the outer half of the VZ (Figure 1L), and *Tead2* is uniformly expressed in the VZ but not in all cells (Figure 1M). *Cdon*, *Celsr1*, *Eomes*, and *Neurog2* (Figures 1B,C,F,H) are expressed more densely in the VZ of the neocortex than in the VZ of the ganglionic eminence (GE; arrow), suggesting that these genes serve a more specific role in neocortical VZ function.

*Neurog2* is involved in generation of glutamatergic cortical neurons and also in repression of GABAergic neuron differentiation during cortical development (Roybon et al., 2010). Consistent with GABAergic neuron repression, *Neurog2* expression is greatly reduced in the GE where GABAergic neurons originate (Figure 1H, arrow). We found that a subset of genes in our VZ network (Figure 1A, box) also have reduced GE expression; *Cdon*, *Celsr1* and *Eomes* (Figures 1B,C,F, arrow). All four genes have reduced GE expression at an earlier stage of development as well (E13.5, data not shown). *Eomes* is known to have a positive role in the migration of the GABAergic neurons from the GE to the SVZ of the neocortex (Sessa et al., 2010). Potentially, expression of *Eomes* in the GE could inhibit the migration of those neurons to the correct location in the SVZ. *Cdon* and *Celsr1* are not known to be involved with GABAergic neuron migration, however, *Celsr1* has a positive role in the migration of facial branchiomotor neurons (Boutin et al., 2012). A role for *Celsr1* and *Cdon* in GABAergic cortical neurons could be investigated based on the expression pattern similarity to *Eomes* and *Neurog2* in the GE.

In addition to VZ expression, we found that six of the VZ network genes (*Dbi*, *Eomes*, *Hmgn2*, *Neurog2*, *Notch1* and *Ssrp1*; Figures 1D,F–I,L) are expressed at low to intermediate levels in regions of the neocortex outside of the VZ. *Dbi* (diazepam binding inhibitor, Figure 1D) is expressed in the VZ and SVZ. Consistent with a role for *Dbi* in the progenitor cells of the neocortex, *Dbi* has been shown to be involved in proliferation of SVZ progenitors in postnatal mice (Alfonso et al., 2012). *Eomes* (also known as *Tbr2*) and *Neurog2* are additionally expressed in the SVZ and intermediate zone (IZ; Figures 1F,H). Both of these genes are transcription factors that regulate cortical progenitor cell maturation (Wilkinson et al., 2013; Sun and Hevner, 2014). *Notch1*, which is known to regulate multiple processes in the neocortex (Sun and Hevner, 2014), has reduced expression in the SVZ and moderate expression in the IZ (Figure 1I). *Ssrp1* and *Hmgn2* are chromatin binding proteins that have not been implicated in cortical development, although *Ssrp1* has been shown to be important in Drosophila neural stem cell self-renewal (Neumüller et al., 2011). We found that both of these genes are highly expressed in the VZ and also show reduced expression in the other regions of the neocortex (Figures 1G,L). Taken together, these six genes are likely involved in multiple steps of cortical development, which has already been demonstrated for three of these genes; *Eomes*, *Neurog2* and *Notch1* (Sun and Hevner, 2014).

### GeneMANIA association network and go analysis: mouse

**Figure 2:**
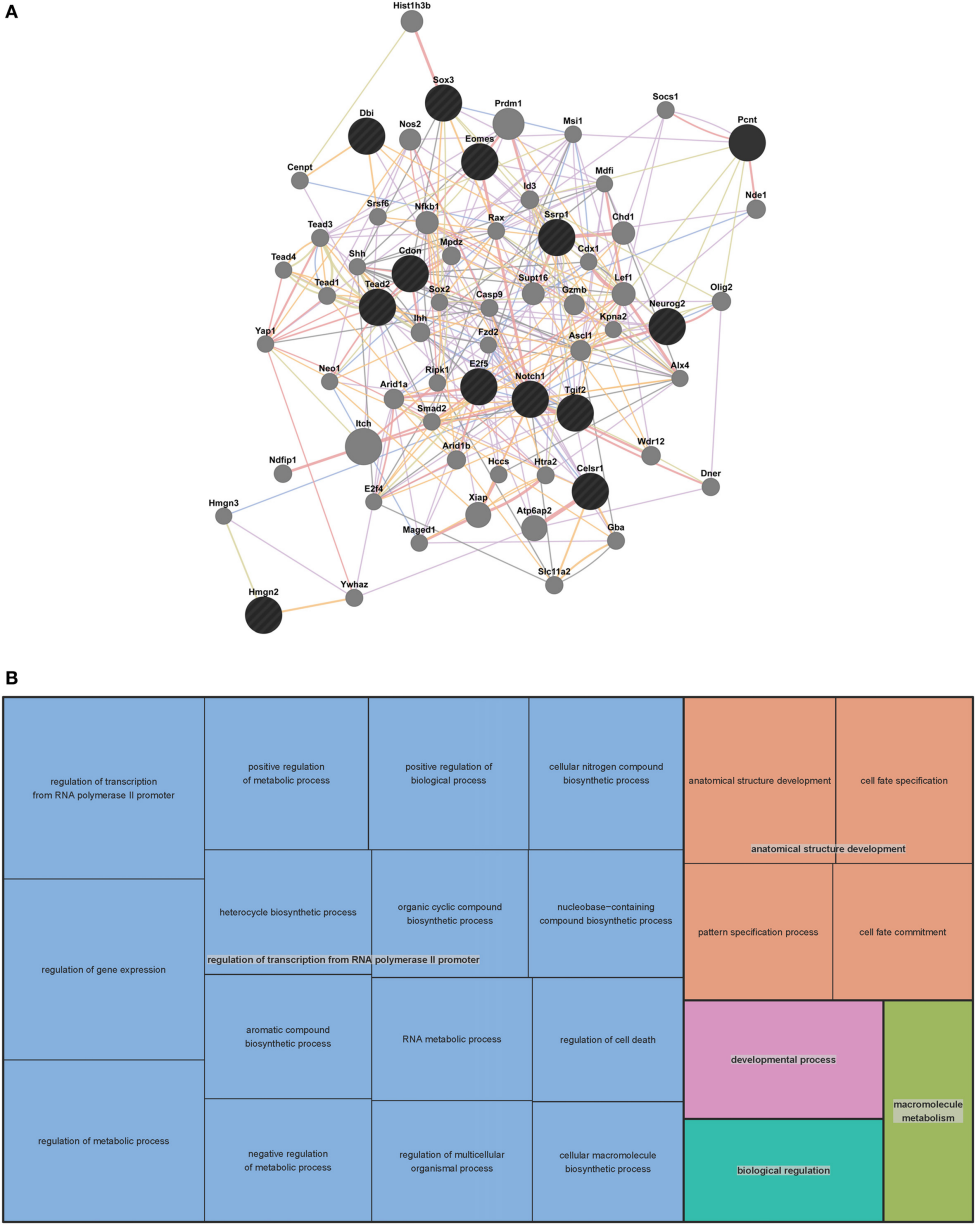
**Mouse extended association analysis network and Gene Ontology (GO) analysis of network genes. (A)** Association analysis of the 13 mouse VZ network genes. 50 associated genes are shown in the extended network. Black circles indicate the genes that were searched through GeneMANIA and the gray circles are associated genes. The edges are indicated by associations found through co-expression (purple lines), co-localization (blue lines), physical interaction (pink lines), predicted (orange lines) and shared protein domains (tan lines). Table S1 contains complete network details and methods. **(B)** “TreeMap” view of the GO analysis of 63 genes from the extended mouse network. Rectangles are cluster representatives, which are joined into superclusters of loosely related GO terms and are represented by different colors. The size of the rectangles is a reflection of the adjusted *p*-value of the enrichment of each GO term relative to the mouse reference list. GO analysis was performed by GOrilla (Eden et al., 2007, 2009) and the “TreeMap” was generated in REViGO (Supek et al., 2011). All GO terms, associated *p*-values and genes for each term can be found in Table S2.

In order to predict potential interactions between the genes in our VZ network and with additional genes outside of the network, we generated an extended mouse network (Figure 2A). We used GeneMANIA (http://genemania.org/), a web-based interface that searches a large set of functional association data to return related genes based on available genomic and proteomic data. The association data include protein, DNA and genetic interactions, pathways, gene and protein expression data, phenotypic screens and shared protein domains (Zuberi et al., 2013). The VZ network gene list of 13 was extended to a list of 63 genes through GeneMANIA (Table S1 contains search parameters, the returned gene list, statistics used, plus additional outputs).

To predict the function of the 63 genes that are enriched in the extended mouse network, we examined GO enrichment of this gene list. We used publically available web-based GO analysis tools, GOrilla (http://cbl-gorilla.cs.technion.ac.il/) and REViGO (http://revigo.irb.hr/). We found a significant enrichment in genes involved in transcriptional regulation, anatomical structure development and macromolecule metabolism (Figure 2B and Table S2). We also evaluated GO enrichment in the original 13 genes from the mouse VZ network (data not shown). GO enrichment is not usually considered statistically significant in small gene lists, however, we did find that 10 of the 13 genes are involved in the regulation of transcription. This analysis demonstrates that the genes in our mouse VZ and extended networks are involved in transcriptional regulation, which is expected for genes implicated in early developmental processes such as neocortical development. In addition, the extended network is validated by common GO enrichment with the VZ network.

### Human developing brain network

**Figure 3:**
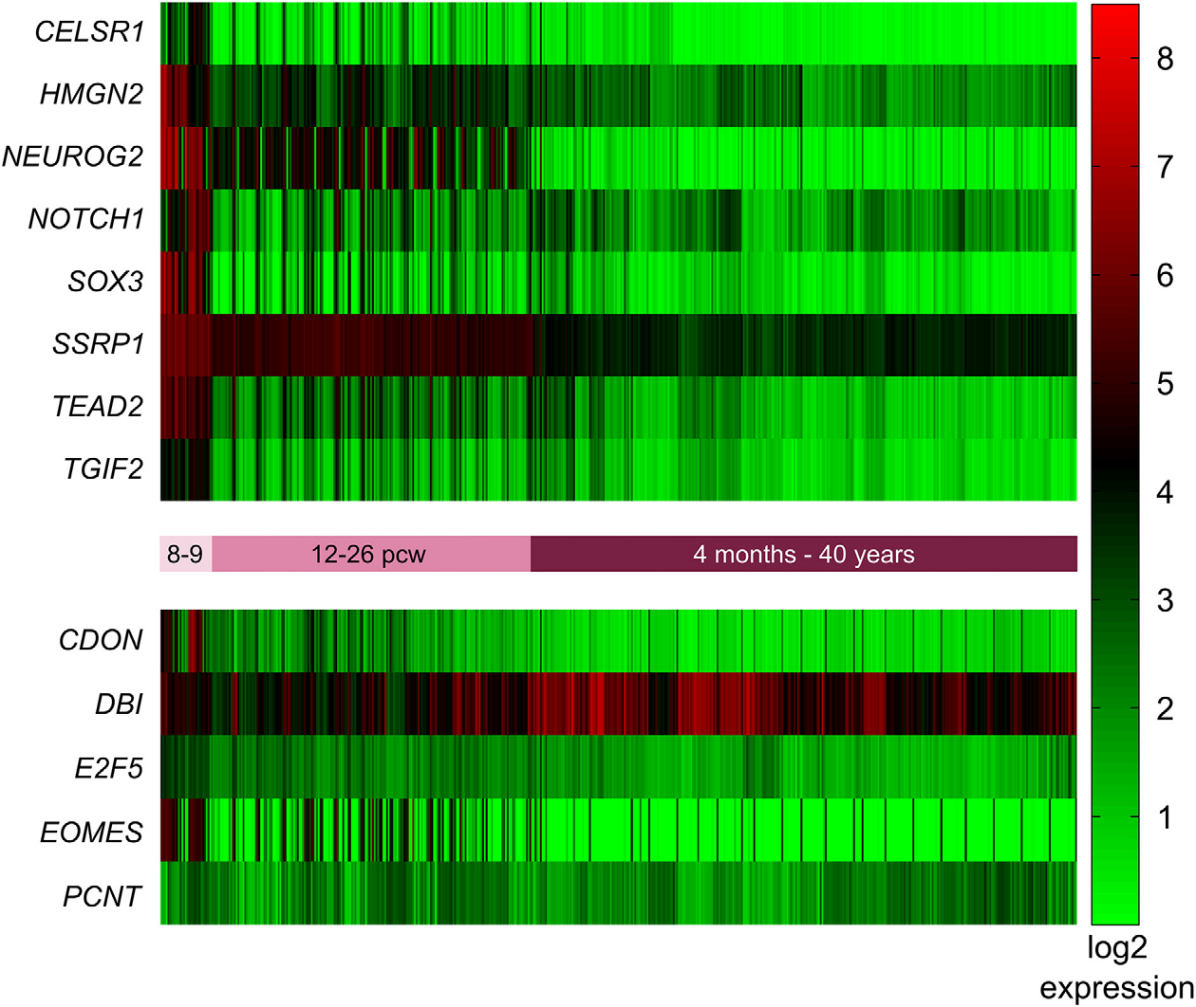
**Expression of mouse VZ network genes in different areas of the human developing brain (15–20 post-conception weeks)**. Genes with significantly higher expression in the ventricular and subventricular zone (VZ and SVZ respectively) compared to all other brain regions were identified using the prenatal laser microdissection (LMD) microarray dataset from the BrainSpan atlas of the developing human brain. Resulting gene lists were then compared to those genes identified in mice. We found that ten of the 13 mouse genes were significantly more highly expressed in the human VZ and SVZ, namely *CDON*, *CELSR1*, *E2F5*, *EOMES*, *HMGN2*, *NEUROG2*, *NOTCH1*, *SOX3*, *TEAD2*, and *TGIF2*. The other three genes—*DBI*, *PCNT*, and *SSRP1*—were not enriched in the human VZ or SVZ. MZ, marginal zone; CP, cortical plate; SP, subplate zone; IZ, intermediate zone; SZ, subventricular zone; VZ, ventricular zone; AMY, amygdaloid complex; THM, thalamus; Tg, tegmentum; NP, neural plate.

To generalize the data from the mouse network we aimed to determine genes with high expression in the VZ and SVZ of the human neocortex early in development. To this end we made use of the BrainSpan Atlas of the developing human brain (http://brainspan.org/). We started by identifying genes with strongest expression in the SVZ and VZ from the BrainSpan LMD microarray dataset (comprised of microarray profiles of samples taken from ~300 different brain structures at 15–21 pcw). Using the web interface of the BrainSpan atlas we identified data from 24,084 probes with at least the same level of expression (i.e., one-fold) in the VZ and SVZ, compared to all other areas. From this list we removed all data points with less than a six-fold difference in expression in the VZ and SVZ compared to all other areas, resulting in a list of data points from 2746 remaining probes. After Holm-Bonferroni correction the *p*-values of all data points remained below the significant *p*-value of 1×10^−9^. This set of 2746 data points contained 1781 different genes. Ten of the genes from this list (*CDON*, *CELSR1*, *E2F5*, *EOMES*, *HMGN2*, *NEUROG2*, *NOTCH1*, *SOX3*, *TEAD2*, *TGIF2*) were among the 13 mouse genes in the network described above (Figure 3).

**Figure 4:**
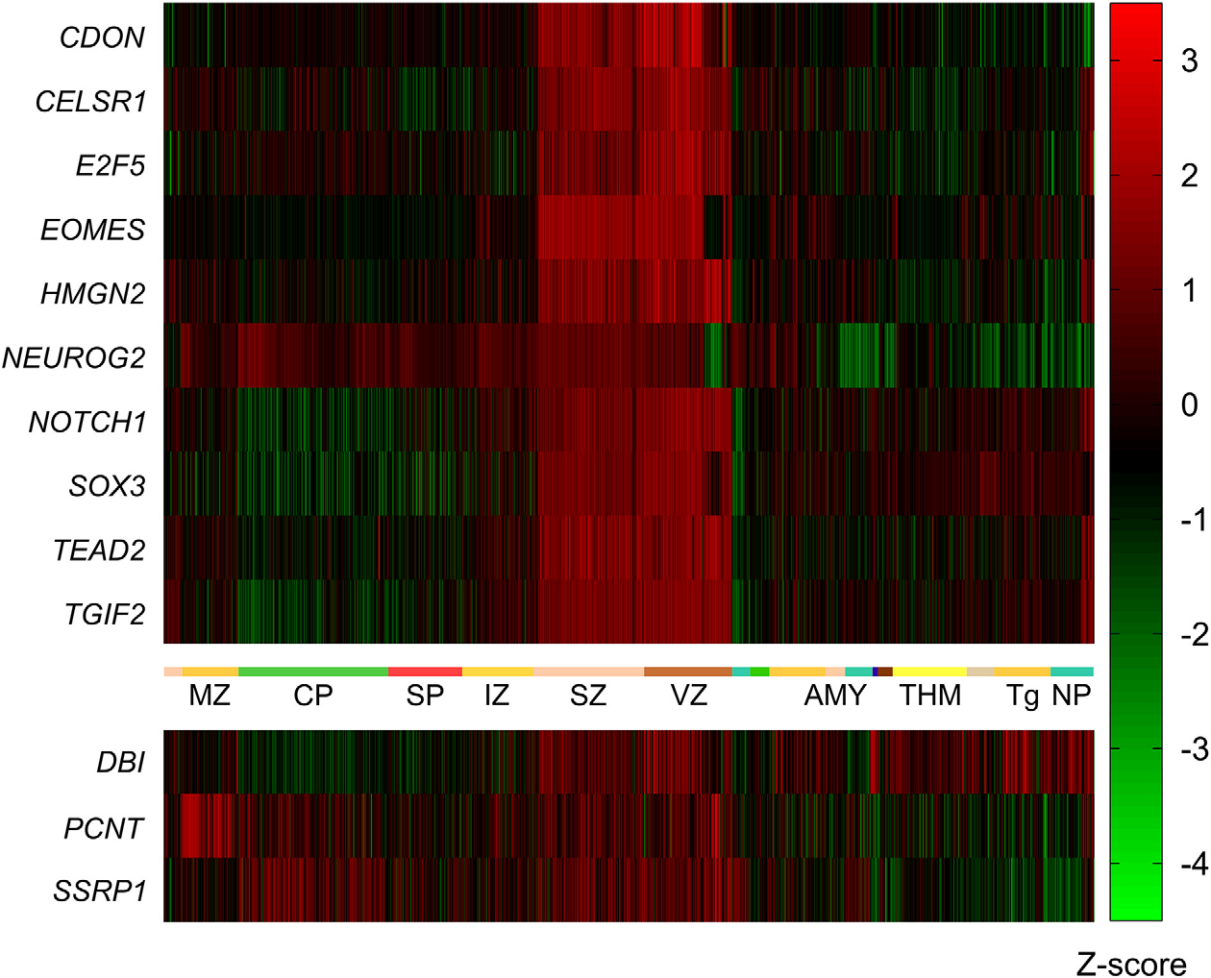
**Expression of mouse network genes in the human brain throughout development**. Genes with significantly higher expression at post-conception weeks (pcw) 8–9 compared to later time points were analyzed using the developmental transcriptome dataset from the BrainSpan atlas of the developing human brain. Eight of the 13 mouse genes were significantly more highly expressed at 8–9 pcw: *CELSR1*, *HMGN2*, *NEUROG2*, *NOTCH1*, *SOX3*, *SSRP1*, *TEAD2*, and *TGIF2*. *CDON*, *DBI*, *E2F5*, *EOMES*, and *PCNT* did not show increased expression during 8–9 pcw compared to later stages.

In addition to identifying the genes with high expression in the SVZ and VZ, we wanted to identify those genes that are highly expressed early in development (pcw 8–9) compared to later time points. Again we employed the BrainSpan web interface to access the transcriptome dataset [comprised of RNA sequencing and exon microarray data from brains at different developmental stages (8 pcw up to 39 years of age)]. Our query resulted in a list of 18,105 genes with = one-fold expression at pcw 8–9 compared to all later stages. This list was narrowed down to 1176 after removing all genes with less than a six-fold expression difference at pcw 8–9 compared to all later stages. After Holm-Bonferroni correction 1048 of these genes remained below the significant *p*-value of 1 × 10^−9^. Eight of these genes (*CELSR1*, *HMGN2*, *NEUROG2*, *NOTCH1*, *SOX3*, *SSRP1*, *TEAD2*, *TGIF2*) were also present in the mouse network described above (Figure 4).

We then compared the resulting gene lists from the LMD microarray dataset and the developmental transcriptome, and found that 407 genes were overlapping between these two lists (Table S3). From these 407 genes, seven (*CELSR1*, *HMGN2*, *NEUROG2*, *NOTCH1*, *SOX3*, *TEAD2*, *TGIF2*) were also present in the mouse VZ network. An additional six genes overlap with the mouse extended network (*FZD2, HIST1H3B, NDE1, SOX2, TEAD1* and *YAP1*). The overlap between these networks is a further validation of the publically available datasets and methods used to generate the networks.

### GeneMANIA association network: human

**Figure 5:**
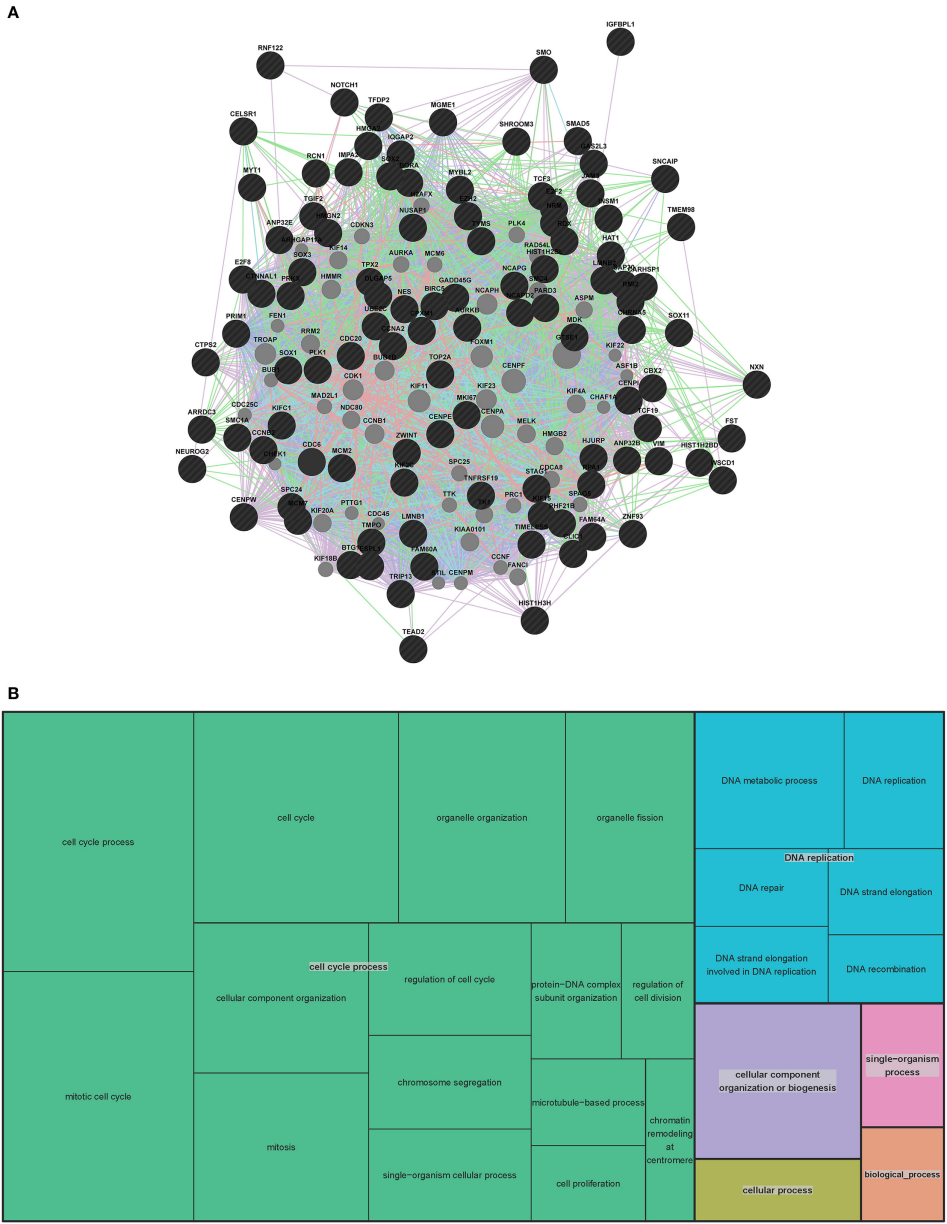
**Human extended association analysis network and Gene Ontology (GO) analysis of human network genes. (A)** The top 100 genes from the human network and the 7 genes that overlap between the human and mouse network were used to search for associations between those genes and to find additional genes that associate with them. 50 associated genes are shown in the network. Black circles indicate the genes that were searched through GeneMANIA and the gray circles are associated genes. The edges are indicated by associations found through co-expression (purple lines), co-localization (blue lines), physical interaction (pink lines) and shared protein domains (tan lines). Predicted associations were not included in the human network due to the large amount of genes. Table S4 contains complete network details and methods. **(B)** “TreeMap” view of the GO analysis of 407 genes that are expressed at early stages of human VZ and SVZ development. Rectangles are cluster representatives, which are joined into superclusters of loosely related GO terms and are represented by different colors. The size of the rectangles are a reflection of the adjusted *p*-value of the enrichment of each GO term relative to the human reference list. GO analysis was performed by GOrilla (Eden et al., 2007, 2009) and the “TreeMap” was generated in REViGO (Supek et al., 2011). All GO terms, associated *p*-values and genes for each term can be found in Table S5.

To further evaluate the genes involved in human VZ and SVZ development, we generated an extended human network using GeneMANIA (Figure 5 and Table S4). Due to the length of the human gene list we used the top 100 human genes and the 7 genes that overlapped with the mouse VZ network to generate the extended network. As with the mouse extended network, we allowed 50 additional genes to be added to create our extended network. Interestingly, 46 of the 50 genes are in the complete list of 407 human genes involved in VZ and SVZ development. This highly substantiates the use of GeneMANIA as a tool for predicting relationships between genes. The four genes that are unique to the extended human network (*MCM6, KIF18B, FEN1* and *CCNF*) are known to be involved in DNA replication, mitosis and cell cycle specific proteasome degradation.

### Pathway and go analysis

To gain insight into the function of the 407 genes that are enriched in the human SVZ and VZ, we examined GO and pathway enrichment of these genes. In addition to GOrilla and REViGO (used for the mouse GO analysis) we also used WebGestalt (http://bioinfo.vanderbilt.edu/webgestalt/), which has several enrichment analysis tools (Eden et al., 2007, 2009; Supek et al., 2011; Wang et al., 2013). We found a significant enrichment in genes involved in the cell cycle, DNA replication and specific stages of mitotis. The top 10 pathways that are enriched in this gene list are represented in Table 1. Among the GO enriched terms for biological processes are cell cycle process, mitotic cell process, DNA replication and chromatin assembly and disassembly (Figure 5B and Table S5). The pathway and GO analysis for the human network is consistent with functions expected for genes involved in early cortical development.

**Table 1:** **Pathway enrichment analysis of genes highly enriched in the ventricular and subventricular zone during early human development using WebGestalt**.

**Pathway name**	**#Genes**	**Statistics**
Cell cycle, Mitotic	96	*C* = 318;*O* = 96; *E* = 2.99; *R* = 32.07; rawP = 6.24e–118; adjP = 2.10e–115
DNA Replication	76	*C* = 261; *O* = 76; *E* = 2.46; *R* = 30.93; rawP = 1.89e–91; adjP = 3.18e–89
Mitotic M-M/G1 phases	69	*C* = 242; *O* = 69; *E* = 2.28; *R* = 30.29; rawP = 3.48e–82; adjP = 3.91e–80
M Phase	46	*C* = 158; *O* = 46; *E* = 1.49; *R* = 30.93; rawP = 3.06e–55; adjP = 2.58e–53
Mitotic prometaphase	39	*C* = 99; *O* = 39; *E* = 0.93; *R* = 41.85; rawP = 4.88e–53; adjP = 3.29e–51
Mitotic G1-G1/S phases	40	*C* = 141; *O* = 40; *E* = 1.33; *R* = 30.13; rawP = 1.33e–47; adjP = 7.47e–46
ATM pathway	45	*C* = 307; *O* = 45; *E* = 2.89; *R* = 15.57; rawP = 1.45e–39; adjP = 6.98e–38
G1/S Transition	32	*C* = 118; *O* = 32; *E* = 1.11; *R* = 28.81; rawP = 1.48e–37; adjP = 6.23e–36
Polo-like kinase signaling events in the cell cycle	31	*C* = 109; *O* = 31; *E* = 1.03; *R* = 30.21; rawP = 3.85e–37; adjP = 1.44e–35
S Phase	31	*C* = 121; *O* = 31; *E* = 1.14; *R* = 27.21; rawP = 1.48e–35; adjP = 4.99e–34

A list of 407 genes was provided to WebGestalt and the predefined human reference genome was used as the reference set. The top 10 most significantly enriched pathways are shown with an adjusted p-value of 4.99 × 10^-*34*^ or lower. C, number of reference genes in category; O, number of genes in the category from the gene set; E, expected number in category; R, ratio of enrichment; rawP, p-value from hypergeometric test; adjP, p-value adjusted by the multiple test adjustment.

## Discussion

### Generation of gene networks that are involved in CNS developmental processes

Development of the CNS is a set of processes that increase the complexity of the brain. Genes are spatially and temporally coordinated to control normal CNS development, while perturbed modulation results in neurological diseases. To understand the regulatory network during these processes, spatio-temporal information of gene expression in the brain is required. In recent decades, spatial mapping of ISH has been a useful tool to demonstrate regional gene expression patterns in different model organisms (De Boer et al., 2009). The Allen Institute for Brain Science provides high resolution ISH datasets containing spatial information at multiple developmental stages of the mouse brain. These datasets allowed us to generate a network composed of correlated genes that are highly expressed in the VZ at early stages of mouse neocortical development (Figure 1). It should be pointed out that only 10% of the mouse genes exist in the Developing Mouse Brain Atlas. Therefore, we clearly significantly underestimate the number of genes involved in mouse VZ development. Even with this underestimation, novel gene regulators were identified based on the derived gene network. As a second example, we generated a non-VZ network of genes expressed in regions adjacent to the VZ at the same stage (Figure S1). Note that all of the gene expression patterns shown by the Allen Developing Mouse Brain Atlas are specific to the C57BL/6J mouse strain and, thus, do not address variation along the genetic axis (Peirce and Williams, 2006).

We further performed a cross-species data search with the BrainSpan Atlas of the Developing Human Brain (Miller et al., 2014), specifically of the VZ and SVZ of the neocortex (Figures 3, 4). The standardized bioinformatics pipeline employed by the Allen Brain Atlas enabled the usage of data from different sources and the combination of data across different species (Sunkin et al., 2013). Translating developmental time between mice and human, we chose comparable time points to analyze the human data that were used earlier to compile the mouse neocortical data (Clancy et al., 2001). The increased sensitivity of the methodology in the Human Developing Atlas, which uses next generation sequencing and microarray technologies, allows identifications of more regulators of neocortical development. This integrated cross-species approach is a powerful tool to fully exploit stored gene expression data, and unravel the gene regulatory networks during brain development with both spatial and temporal information.

### Identification of novel gene regulators and functions during neocortical development

The growth of the cerebral cortex largely depends on the proliferation of neural progenitors, and the subsequent generation of differentiated neural cells. Consistent with this, GO analysis of the mouse VZ network and extended network suggests involvement in cell fate specification and commitment, regulation of transcription and developmental processes. For example, *Notch1* regulates cell cycle progression, which maintains the stem population in both the subgranular zone of the dentate gyrus and the SVZ (Aguirre et al., 2010; Ables et al., 2011). The acquisition of radial glia and astrocytes with stem cell properties is also promoted by *Notch1* (Gaiano et al., 2000), while neuronal and oligodendrocyte differentiation are inhibited (Wang et al., 1998; Yoon and Gaiano, 2005). Later in development, altered expression levels of *Notch1* affect the morphology of neurons, and perturb the neuronal migration in the cerebral cortex (Hashimoto-Torii et al., 2008). *Sox3*, another candidate gene, has a function during brain development similar to that of *Notch1*. Notably, several studies performed in non-mammalian species indicate that *Sox3* negatively regulates neurogenesis to maintain the stemness of precursors (Bylund et al., 2003; Archer et al., 2011).

Some of our candidate genes are known to be involved in neurogenesis and neuronal migration, which are essential processes in the development of the cerebral cortex. *Neurog2* is a proneural transcription factor initiating neuronal differentiation (Wilkinson et al., 2013). When overexpressed *Neurog2* promotes neurogenesis more rapidly in dorsal vs. ventral telencephalic progenitor cells specifically at E12.5 in mice (Li et al., 2012a), suggesting the modulation of *Neurog2* is spatially and temporally regulated. Interestingly, *Neurog2* is one of the four genes (*Cdon*, *Celsr1*, *Eomes*, and *Neurog2*) specifically reduced in the mouse GE compared with the VZ of the neocortex (Figures 1B,C,F,H). The GE emerges early in telencephalic development. Cells from the GE migrate tangentially and supply inhibitory neurons (i.e., GABAergic neurons) to the neocortex. Indeed, *Neurog2* represses the generation of the GABAergic neuronal phenotype whereas it promotes the generation of glutamatergic neurons when facing the binary cell fate decision (Schuurmans et al., 2004). Another gene that we found to be downregulated in the mouse GE is *Eomes*, which we also found to be specifically expressed in the human VZ and SVZ early in development (however note that expression of *EOMES* 8–9 pcw compared to all later stages was only nominally significant and did not survive correction for multiple testing) (Figures 3, 4). Similar to *Neurog2*, *Eomes* promotes neuronal-fate commitment (Hodge et al., 2012). Studies demonstrate that *Eomes* might be required to control the equilibrium between glutamatergic and GABAergic neurons that migrate either to the olfactory bulb (Mizuguchi et al., 2012) or cerebral cortex (Sessa et al., 2010). *Celsr1* is implicated in both tangential and radial migration as well, in facial branchiomotor neurons (Boutin et al., 2012). Hence, our results imply that *Cdon* and *Celsr1* are likely to be involved in regulating the size of the excitatory-inhibitory neuronal pool.

Only limited data is available for the other candidate genes (*Tead2*, *Tgif2*, *Cdon*, *Dbi*, *Pcnt*, *E2f5*, *Hmgn2*, and *Ssrp1*) during early neural development. *Tead2* is a transcription factor that is essential for neural tube closure (Kaneko et al., 2007), probably via regulating cell proliferation and apoptosis (Sawada et al., 2008). The contribution from *Tgif2* in holoprosencephaly (failure of the embryonic forebrain to completely divide into two hemispheres) is still controversial (Shen and Walsh, 2005; Taniguchi et al., 2012). Notably, other genes not in our combined human and mouse candidate list, including *Cdon*, *Dbi* and *Pcnt*, also exhibit potential effects during neural development (Zhang et al., 2006; Oh et al., 2009; Buchman et al., 2010; Endoh-Yamagami et al., 2010; Alfonso et al., 2012). Loss of *E2f5* does not perturb cell proliferation in the VZ during early development in mice (Lindeman et al., 1998), which might be due to the compensating effect of other E2F transcription factors (e.g., *E2f1*) (Yoshikawa, 2000; Cooper-Kuhn et al., 2002). *Hmgn2* and *Ssrp1* are both chromatin binding proteins that are novel potential regulators of neocortical development implicated by our examination of a publically available dataset. This is also supported by their involvement in DNA replication and cell cycle progression (Cherukuri et al., 2008; Formosa, 2012).

### Validation of GeneMANIA generated network by literature mining

**Figure 6:**
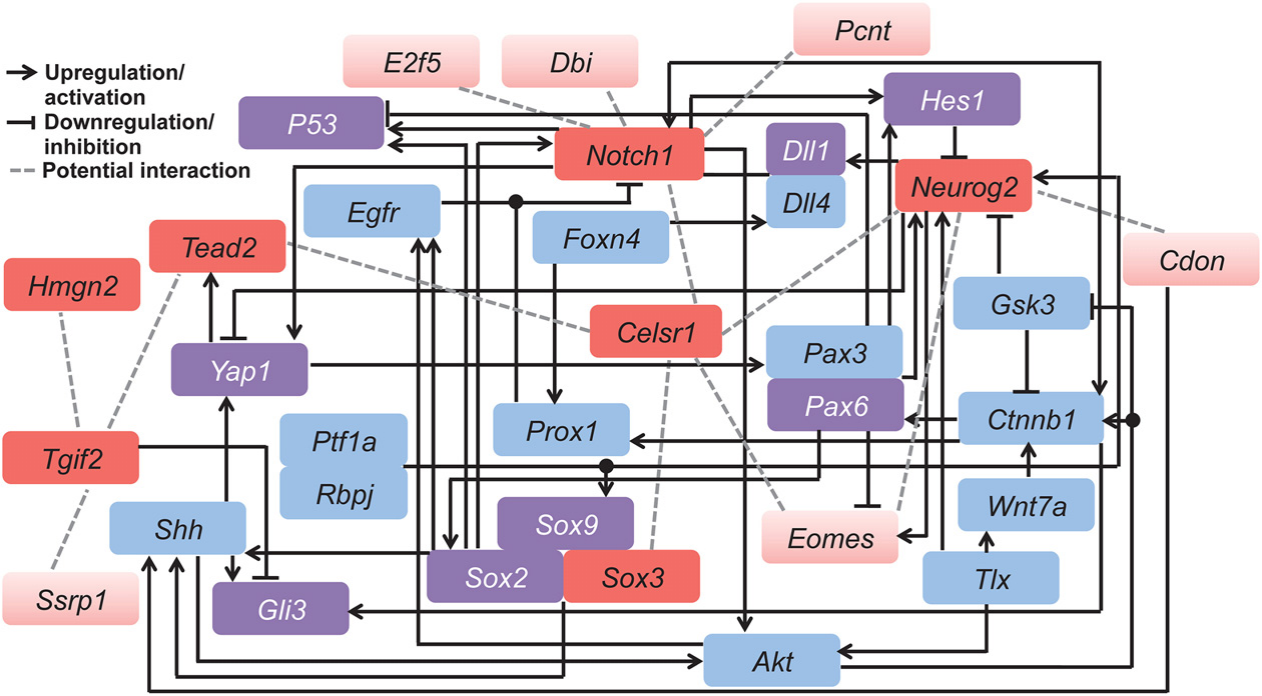
**Hypothetical functional gene network involved in regulating neocortical development**. This model was derived by combining our candidate genes (red) with related genes (blue and purple) known from published mammalian cell line or animal studies. Among all candidates, the seven genes conserved in both the mouse and human networks are indicated in dark red, and those specific to the mouse network are in light red. Purple color indicates genes that overlap with the 407 genes from the human developing brain network. Different types of gene interactions are indicated by (1) black arrows: upregulation or activation; (2) black lines with flat end: downregulation or inhibition; (3) gray dashed lines: potential interaction. Table S6 contains details of the literature mining methods.

Potential cross-talk among our candidate genes was verified by literature mining. We made our space-time VZ network (Figure 1) into a more complex and hypothetical functional gene network (Figure 6). In line with GeneMANIA analysis, *Neurog2*, *Eomes* and *Notch1* closely interact with each other. In fact, *Eomes* and *Neurog2* have similar expression patterns in the cortical progenitors of the VZ, which is initiated by direct binding of NEUROG2 to the promoter of *Eomes* (Ochiai et al., 2009). In addition, these two genes interact via co-regulators of transcription. *Pax6* negatively regulates the expression of *Eomes* (Imamura and Greer, 2013), while upregulating *Neurog2* during early neural development (Scardigli, 2003). As mentioned earlier, *Notch1* exhibits opposite effects in neurogenesis compared with *Neurog2* and *Eomes*. Consistently, the *Neurog2*-*Eomes* cascade is suppressed by Notch signaling through *Hes1* with a positive feedback from *Neurog2* via the Notch ligand *Dll1* (Kageyama et al., 2008). The correlation between these genes is also facilitated by the canonical Wnt signaling pathway (Shimizu et al., 2008; Li et al., 2012a). Coordinated interplay among the genes in the network controls the switch between neural stem cells and fate-committed neurons, balancing the pool of each neural population during development.

In contrast, *Hmgn2* displays little interaction with other genes, also shown by the GeneMANIA network (Figure 2A). This further supports the hypothesis that *Hmgn2* is a novel regulator in this network. In addition, some connections indicated by our hypothetical functional gene network are novel. For instance, *Celsr1* shows little interaction with other candidates in previous research. However, it is a central component in our mouse Neocortex VZ Network, and its involvement is conserved in the human network. The interplay between *Celsr1* and other genes are also predicted in both the mouse and human extended networks (Figures 2A, 5A). Thus, *Celsr1* could be a promising target for future neural developmental research. In addition, four of the 28 genes from the hypothetical network overlap with the GeneMANIA extended mouse network (i.e., *Shh*, *Smad2*, *Sox2*, and *Yap1*) (Tables S1,S6), and eight genes overlap with the 407 genes from the human developing brain network (i.e., *DLL1*, *GLI3*, *HES1*, *TP53*, *PAX6*, *SOX2*, *SOX9*, and *YAP1*) (Tables S4,S6). The consistency between the literature mining and GeneMANIA results validates that GeneMANIA can be a powerful tool in unmasking potential genetic interactions.

Taken together, our proposed gene networks provide a comprehensive view to discover genes involved in complex developmental processes, to reveal the relationships among them, and to help understand genetic modulation at a systematic level. Specifically, through the use of databases that incorporate multiple datasets from both human and mouse studies (Allen Brain Atlas and GeneMANIA) and the analysis of the extensive literature on the developing neocortex, we have uncovered at least two genes (*Hmgn2* and *Ssrp1*) from our mouse network not previously known to be involved in neocortical development. In addition, the data from the human indicates 100's of genes potentially involved in ventricular zone development that could be the basis for future investigations.

### Conflict of interest statement

The authors declare that the research was conducted in the absence of any commercial or financial relationships that could be construed as a potential conflict of interest.
