# Supplementary material for: A multi-resource data integration approach: identification of candidate genes regulating cell proliferation during neocortical development
Source: Front Neurosci. 2014 Aug 21;8:257. doi: 10.3389/fnins.2014.00257 (PMC4139594; doi:10.3389/fnins.2014.00257)
Supplement: Supplementary file 1 [file DataSheet1.ZIP › 108271_Nowakowski_Table_3.DOCX]

**Table S1: Expression values for all human genes that are significantly expressed early in development in the ventricular and subventricular zone.** Genes that are in the mouse VZ network are in red and the mouse extended network are in blue.

|  | LMD Microarray | | Transcriptome | |
| --- | --- | --- | --- | --- |
| **Gene symbol** | Corrected p-value | Log2 expression | Corrected p-value | Log2-expression |
| ACTL6A | 3,7397E-13 | 4,591 | 1,3966E-12 | 5,319 |
| ANP32B | 1,0194E-12 | 3,193 | 3,012E-12 | 3,335 |
| ANP32E | 4,4783E-13 | 3,709 | 2,445E-12 | 4,021 |
| ARHGAP11A | 5,168E-13 | 9,342 | 1,2887E-12 | 7,333 |
| ARID2 | 1,1442E-12 | 3,736 | 1,5432E-12 | 2,7 |
| ARL6IP6 | 5,102E-13 | 3,521 | 1,5015E-12 | 4,827 |
| ARRDC3 | 1,3908E-12 | 3,626 | 1,1351E-12 | 7,044 |
| ASAP3 | 4,9285E-13 | 3,262 | 6,4935E-12 | 2,66 |
| ASF1B | 4,0437E-13 | 10,393 | 1,3643E-12 | 6,733 |
| ASPM | 3,8715E-13 | 29,917 | 1,6978E-12 | 8,287 |
| ATAD2 | 4,0064E-13 | 9,271 | 2,2573E-12 | 5,964 |
| AURKA | 3,8971E-13 | 9,621 | 1,7762E-12 | 6,247 |
| AURKB | 5,2138E-13 | 10,383 | 1,1025E-12 | 21,524 |
| BARD1 | 5,277E-13 | 3,737 | 1,3514E-12 | 3,181 |
| BAZ1A | 4,1477E-13 | 4,203 | 1,8657E-12 | 3,975 |
| BCOR | 1,0288E-12 | 3,659 | 3,8023E-12 | 3,211 |
| BIRC5 | 3,6941E-13 | 20,236 | 1,0504E-12 | 21,15 |
| BLM | 1,2531E-12 | 3,375 | 4,1841E-12 | 2,709 |
| BMF | 5,767E-13 | 4,045 | 1,3423E-12 | 2,652 |
| BORA | 4,8426E-13 | 7,807 | 2,5E-12 | 3,128 |
| BRCA1 | 3,7341E-13 | 17,101 | 1,9305E-12 | 3,911 |
| BRIP1 | 2,008E-12 | 3,817 | 2,0202E-12 | 4,809 |
| BTBD17 | 1,1737E-12 | 5,706 | 2,8653E-12 | 3,455 |
| BTG1 | 5,2798E-13 | 3,805 | 1,675E-12 | 2,686 |
| BTG3 | 4,1305E-13 | 4,868 | 2,0492E-12 | 5,197 |
| BUB1 | 3,775E-13 | 16,227 | 1,2438E-12 | 10,3 |
| BUB1B | 4,5496E-13 | 6,59 | 1,2438E-12 | 10,3 |
| C10orf114 | 6,5703E-13 | 3,722 | 5,0251E-12 | 2,972 |
| C15orf42 | 3,9417E-13 | 14,108 | 5,2632E-12 | 3,744 |
| C18orf54 | 8,4962E-13 | 2,768 | 1,5699E-12 | 2,957 |
| C18orf56 | 4,5683E-13 | 4,89 | 1,7123E-12 | 4,14 |
| C20orf72 | 1,046E-12 | 4,079 | 1,1403E-12 | 4,352 |
| CARHSP1 | 6,5833E-13 | 2,816 | 9,6712E-13 | 3,738 |
| CASC5 | 4,662E-13 | 16,554 | 2,7548E-12 | 5,002 |
| CASP6 | 4,6339E-13 | 4,526 | 6,8966E-12 | 2,665 |
| CBX2 | 4,7081E-13 | 4,659 | 1,1186E-12 | 6,96 |
| CCDC8 | 5,3533E-13 | 5,198 | 1,6807E-12 | 4,17 |
| CCNA2 | 3,8447E-13 | 22,738 | 1,1534E-12 | 17,034 |
| CCNB1 | 4,4267E-13 | 6,555 | 1,3441E-12 | 8,002 |
| CCNB2 | 3,6955E-13 | 23,327 | 1,0373E-12 | 20,313 |
| CDC20 | 3,8081E-13 | 22,816 | 1,1364E-12 | 17,252 |
| CDC25A | 6,7159E-13 | 3,624 | 1,3699E-12 | 6,241 |
| CDC25B | 8,1833E-13 | 3,242 | 3,9063E-12 | 3,134 |
| CDC25C | 3,9401E-13 | 12,132 | 1,7331E-12 | 5,066 |
| CDC45 | 3,9683E-13 | 16,827 | 1,4728E-12 | 7,36 |
| CDC6 | 3,9635E-13 | 11,469 | 2,71E-12 | 4,383 |
| CDCA2 | 3,6563E-13 | 24,333 | 1,6529E-12 | 6,688 |
| CDCA3 | 5,0277E-13 | 6,866 | 1,5152E-12 | 6,892 |
| CDCA4 | 4,2052E-13 | 3,902 | 1,2195E-12 | 6,539 |
| CDCA5 | 3,7327E-13 | 10,528 | 1,3532E-12 | 7,611 |
| CDCA7 | 3,7175E-13 | 13,798 | 1,2821E-12 | 12,986 |
| CDCA8 | 4E-13 | 12,631 | 1,2723E-12 | 11,285 |
| CDK1 | 3,864E-13 | 21,282 | 1,2674E-12 | 14,763 |
| CDK2 | 3,7369E-13 | 3,093 | 1,2077E-12 | 8,462 |
| CDK4 | 4,2499E-13 | 2,739 | 2,4331E-12 | 3,755 |
| CDK6 | 5,2687E-13 | 5,334 | 1,9048E-12 | 4,701 |
| CDKN2C | 3,6873E-13 | 21,355 | 6,8027E-12 | 3,304 |
| CDKN3 | 4,0032E-13 | 10,401 | 2,7473E-12 | 4,478 |
| CDT1 | 4,3917E-13 | 9,126 | 1,3495E-12 | 9,24 |
| **CELSR1** | **3,8095E-13** | **17,916** | **5,7803E-12** | **6,213** |
| CENPA | 3,6711E-13 | 27,678 | 1,6393E-12 | 5,358 |
| CENPE | 5,3362E-13 | 6,207 | 4,902E-12 | 3,588 |
| CENPF | 4,0048E-13 | 10,857 | 1,6694E-12 | 11,697 |
| CENPH | 4,1841E-13 | 3,161 | 1,1765E-12 | 5,806 |
| CENPI | 5,2604E-13 | 5,881 | 2,381E-12 | 3,411 |
| CENPK | 4,6361E-13 | 7,518 | 2,7322E-12 | 3,748 |
| CENPL | 7,5873E-13 | 3,158 | 3,4247E-12 | 2,658 |
| CENPM | 3,8402E-13 | 11,191 | 1,6207E-12 | 6,178 |
| CENPN | 3,8153E-13 | 10,595 | 3,7453E-12 | 2,88 |
| CENPQ | 5,4377E-13 | 6,008 | 7,5758E-12 | 3,095 |
| CENPW | 7,1736E-13 | 2,621 | 2,907E-12 | 5,402 |
| CEP55 | 3,8226E-13 | 15,382 | 1,7065E-12 | 6,633 |
| CHAF1A | 9,9502E-13 | 2,596 | 2,1142E-12 | 4,06 |
| CHAF1B | 6,3776E-13 | 2,99 | 2,1231E-12 | 2,916 |
| CHD7 | 3,9078E-13 | 5,446 | 1,4641E-12 | 5,05 |
| CHEK1 | 4,717E-13 | 5,834 | 1,7606E-12 | 5,674 |
| CHEK2 | 4,0161E-13 | 10,178 | 2,4876E-12 | 2,872 |
| CHRNA5 | 1,2107E-12 | 2,783 | 1,692E-12 | 2,738 |
| CHST14 | 3,9124E-13 | 5,727 | 3,0211E-12 | 3,026 |
| CKAP2 | 3,7411E-13 | 15,885 | 1,4535E-12 | 8,111 |
| CKAP2L | 3,7411E-13 | 15,885 | 1,4535E-12 | 8,111 |
| CKS1B | 4,3898E-13 | 4,701 | 2,4096E-12 | 4,695 |
| CKS1BP6 | 4,8733E-13 | 4,372 | 2,4096E-12 | 4,695 |
| CKS2 | 4,4743E-13 | 5,687 | 1,4903E-12 | 11,657 |
| CLGN | 8,4674E-13 | 3,028 | 5,0761E-12 | 2,851 |
| CLIC1 | 3,6887E-13 | 5,608 | 1,1574E-12 | 5,119 |
| CMTM3 | 5,6306E-13 | 4,153 | 1,9417E-12 | 2,847 |
| CNN3 | 5,3967E-13 | 3,038 | 1,6026E-12 | 2,653 |
| CNTLN | 4,4053E-13 | 5,618 | 3,8462E-12 | 3,315 |
| COL11A1 | 4,7893E-13 | 10,885 | 7,1429E-12 | 3,629 |
| COL5A1 | 3,9683E-12 | 3,85 | 6,0606E-12 | 2,643 |
| CPNE3 | 4,6318E-13 | 2,832 | 6,9444E-12 | 2,855 |
| CPXM1 | 6,2267E-13 | 4,601 | 8,9847E-13 | 5,059 |
| CRB2 | 1,2077E-12 | 3,687 | 2,3148E-12 | 3,877 |
| CTNNAL1 | 4,4209E-13 | 7,421 | 3,268E-12 | 4,444 |
| CTPS2 | 1,0256E-12 | 2,789 | 8,7108E-13 | 3,15 |
| CXCR4 | 6,9109E-13 | 4,26 | 1,5106E-12 | 5,222 |
| DACH1 | 7,5529E-13 | 5,971 | 1,7301E-12 | 8,075 |
| DBF4 | 4,6019E-13 | 3,95 | 3,0769E-12 | 3,157 |
| DCBLD2 | 2,8011E-12 | 2,614 | 1,3793E-12 | 2,737 |
| DEPDC1 | 4,0193E-13 | 7,697 | 1,5898E-12 | 7,169 |
| DEPDC1B | 4,0193E-13 | 7,697 | 1,5898E-12 | 7,169 |
| DHFR | 4,2863E-13 | 4,568 | 3,2573E-12 | 4,203 |
| DIAPH3 | 6,1087E-13 | 5,008 | 4,8309E-12 | 2,782 |
| DLGAP5 | 3,655E-13 | 26,238 | 1,1628E-12 | 14,72 |
| DLL1 | 5,0813E-13 | 4,32 | 1,1933E-12 | 5,342 |
| DNA2 | 1,7422E-12 | 3,206 | 2,4691E-12 | 3,46 |
| DNMT3B | 3,8314E-12 | 3,176 | 1,5625E-12 | 3,468 |
| DSN1 | 4,9188E-13 | 4,976 | 3,6765E-12 | 3,182 |
| DTL | 4,0816E-13 | 11,785 | 1,5337E-12 | 8,569 |
| DUSP16 | 7,148E-13 | 3,085 | 5,5249E-12 | 3,262 |
| E2F1 | 3,8462E-13 | 13,437 | 2,2936E-12 | 5,154 |
| E2F2 | 3,7707E-13 | 21,322 | 2,079E-12 | 6,013 |
| E2F7 | 3,7979E-13 | 26,954 | 2,2624E-12 | 3,955 |
| E2F8 | 3,6792E-13 | 37,091 | 4,065E-12 | 3,582 |
| ECT2 | 4,5872E-13 | 6,779 | 1,5267E-12 | 5,975 |
| EFNA4 | 4,7438E-13 | 6,344 | 2,4752E-12 | 3,621 |
| EFNB1 | 4,7103E-13 | 3,401 | 1,5576E-12 | 10,582 |
| EFS | 4,2409E-13 | 4,139 | 1,6779E-12 | 2,688 |
| ENOSF1 | 2,1645E-12 | 2,937 | 2,4155E-12 | 3,521 |
| EPHB4 | 6,8966E-13 | 4,251 | 1,7036E-12 | 3,24 |
| ERBB2 | 5,8962E-13 | 4,178 | 1,3072E-12 | 3,449 |
| ERCC6L | 3,8314E-13 | 22,625 | 2,1008E-12 | 3,797 |
| ESCO2 | 4,0323E-13 | 11,506 | 2,7027E-12 | 3,558 |
| ESPL1 | 3,7467E-13 | 17,985 | 4E-12 | 4,776 |
| EXO1 | 3,9463E-13 | 12,582 | 1,7513E-12 | 5,973 |
| EZH2 | 3,7037E-13 | 3,734 | 9,4429E-13 | 7,991 |
| FAM111B | 8,6957E-13 | 6,677 | 2,4814E-12 | 4,87 |
| FAM60A | 3,8521E-13 | 4,292 | 9,6805E-13 | 8,485 |
| FAM64A | 4,1563E-13 | 13,968 | 1,1123E-12 | 20,083 |
| FAM83D | 3,7836E-13 | 16,315 | 1,5601E-12 | 8,158 |
| FANCD2 | 3,9904E-13 | 7,902 | 1,8018E-12 | 4,037 |
| FANCI | 3,8565E-13 | 8,051 | 1,8382E-12 | 5,462 |
| FAT1 | 6,2854E-13 | 4,165 | 3,6364E-12 | 3,392 |
| FBLN1 | 1,4556E-12 | 2,688 | 3,5587E-12 | 3,183 |
| FBXL7 | 5,1921E-13 | 5,115 | 3,6232E-12 | 3,823 |
| FBXO5 | 4,279E-13 | 6,664 | 1,6892E-12 | 6,343 |
| FKBP10 | 4,3687E-13 | 6,76 | 2,9155E-12 | 2,753 |
| FLNA | 1,3004E-12 | 4,04 | 5,4054E-12 | 3,054 |
| FOXJ1 | 2,7548E-12 | 6,667 | 5,1813E-12 | 3,063 |
| FOXM1 | 3,8805E-13 | 13,863 | 1,2092E-12 | 11,81 |
| FST | 5,0454E-13 | 5,909 | 1,1641E-12 | 3,09 |
| FSTL1 | 5,0454E-13 | 5,909 | 1,1641E-12 | 3,09 |
| FZD2 | 3,6576E-13 | 28,078 | 2,4272E-12 | 11,142 |
| FZD8 | 3,6617E-13 | 23,837 | 6,6225E-12 | 7,821 |
| GADD45G | 4,4464E-13 | 9,342 | 2,994E-12 | 3,731 |
| GAS1 | 4,6948E-13 | 11,897 | 6,7568E-12 | 6,944 |
| GAS2L3 | 4,5086E-13 | 7,573 | 4,386E-12 | 3,533 |
| GINS1 | 7,3529E-12 | 2,66 | 1,4663E-12 | 4,653 |
| GINS2 | 4,2571E-13 | 6,532 | 1,2594E-12 | 8,51 |
| GINS3 | 4,9975E-13 | 3,819 | 2,3923E-12 | 2,925 |
| GINS4 | 5,2083E-13 | 7,502 | 2,3529E-12 | 3,494 |
| GLI2 | 3,8329E-13 | 16,176 | 3,1646E-12 | 4,671 |
| GLI3 | 3,7355E-13 | 48,976 | 5,2356E-12 | 8,765 |
| GMNN | 4,2589E-13 | 6,766 | 4,6512E-12 | 4,088 |
| GNG5 | 3,867E-13 | 4,1 | 1,9268E-12 | 2,806 |
| GPC4 | 4,4444E-13 | 9,918 | 2,5907E-12 | 9,25 |
| GPC6 | 6,2814E-13 | 4,112 | 2,0367E-12 | 4,701 |
| GPX8 | 4,1051E-13 | 9,149 | 2,2472E-12 | 3,736 |
| GSG2 | 4,0177E-13 | 13,621 | 2,6667E-12 | 4,596 |
| GTSE1 | 3,7216E-13 | 20,509 | 1,1976E-12 | 9,6 |
| H2AFX | 4,6577E-13 | 5,021 | 2,0161E-12 | 6,895 |
| HAT1 | 7,5245E-13 | 3,25 | 5,988E-12 | 2,601 |
| HAUS8 | 4,8709E-13 | 4,062 | 4,6296E-12 | 3,06 |
| HELLS | 4,2337E-13 | 10,054 | 1,7575E-12 | 4,329 |
| HES1 | 4,8146E-13 | 7,897 | 3,1949E-12 | 7,879 |
| HES6 | 4,4524E-13 | 5,47 | 1,1723E-12 | 4,716 |
| HIST1H1C | 3,8926E-13 | 7,099 | 2,0284E-12 | 3,289 |
| HIST1H1D | 4,3141E-13 | 11,258 | 4,6948E-12 | 2,849 |
| HIST1H2AE | 7,7101E-13 | 4,104 | 2,5707E-12 | 3,248 |
| HIST1H2BD | 4,8473E-13 | 7,46 | 3,663E-12 | 4,244 |
| HIST1H2BG | 4,9875E-13 | 6,832 | 3,7037E-12 | 4,414 |
| HIST1H2BH | 5,6275E-13 | 5,715 | 3,2468E-12 | 6,196 |
| HIST1H2BJ | 4,2283E-13 | 8,045 | 3,0488E-12 | 2,878 |
| HIST1H2BL | 5,8928E-13 | 5,329 | 5,102E-12 | 2,747 |
| HIST1H2BM | 6,0386E-13 | 4,588 | 4,8544E-12 | 2,631 |
| HIST1H3B | 4,2937E-13 | 13,13 | 6,7114E-12 | 4,148 |
| HIST1H3H | 8,2713E-13 | 5,911 | 5E-12 | 2,782 |
| HIST2H2AC | 4,1511E-13 | 8,57 | 5,1282E-12 | 3,218 |
| HIST2H2BE | 4,845E-13 | 6,334 | 2,1322E-12 | 2,974 |
| HJURP | 5,2826E-13 | 10,706 | 1,321E-12 | 8,954 |
| HMGA2 | 1,2594E-12 | 5,785 | 5,814E-12 | 8,253 |
| HMGB2 | 3,7807E-13 | 16,017 | 1,1792E-12 | 16,282 |
| **HMGN2** | **4,8972E-13** | **2,812** | **1,4749E-12** | **6,747** |
| HMMR | 3,8551E-13 | 10,826 | 1,5552E-12 | 4,77 |
| ID4 | 4,1736E-13 | 9,759 | 1,6051E-12 | 8,285 |
| IGFBPL1 | 8,8106E-13 | 4,074 | 9,3458E-13 | 15,526 |
| IGSF9 | 8,1433E-13 | 2,655 | 2,3095E-12 | 4,732 |
| IMPA2 | 3,9714E-13 | 8,235 | 6,25E-12 | 2,837 |
| INHBB | 3,6751E-13 | 11,697 | 7,0922E-12 | 3,22 |
| INSM1 | 4,2753E-13 | 8,795 | 1,0173E-12 | 7,761 |
| IQGAP2 | 3,9047E-13 | 20,091 | 6,135E-12 | 4,529 |
| IQGAP3 | 5,322E-13 | 10,842 | 2,4213E-12 | 5,265 |
| ITGB8 | 8,2372E-13 | 5,578 | 3,5971E-12 | 3,492 |
| ITPRIP | 6,402E-13 | 6,147 | 2,2523E-12 | 3,549 |
| JAM3 | 6,8074E-13 | 3,249 | 1,1211E-12 | 2,787 |
| KDELC1 | 5,2743E-13 | 2,607 | 1,6447E-12 | 2,705 |
| KIAA0101 | 3,7679E-13 | 17,963 | 1,5456E-12 | 10,224 |
| KIAA0922 | 8,9606E-13 | 4,267 | 2,3202E-12 | 3,776 |
| KIF11 | 3,7693E-13 | 16,696 | 1,1876E-12 | 15,482 |
| KIF14 | 1,5456E-12 | 4,211 | 2,4631E-12 | 3,613 |
| KIF15 | 3,6603E-13 | 14,545 | 1,385E-12 | 10,466 |
| KIF18A | 3,69E-13 | 18,381 | 1,6611E-12 | 5,311 |
| KIF20A | 4,0469E-13 | 18,399 | 1,2626E-12 | 11,477 |
| KIF20B | 6,0423E-13 | 3,429 | 1,8904E-12 | 3,625 |
| KIF22 | 9,5057E-13 | 2,64 | 1,2255E-12 | 5,286 |
| KIF23 | 3,8139E-13 | 12,724 | 1,5848E-12 | 6,686 |
| KIF2C | 3,7092E-13 | 19,056 | 1,1521E-12 | 12,925 |
| KIF4A | 3,9339E-13 | 13,808 | 1,2346E-12 | 8,18 |
| KIFC1 | 3,873E-13 | 26,811 | 1,1287E-12 | 15,325 |
| KNTC1 | 4,6211E-13 | 5,387 | 2,7778E-12 | 3,732 |
| LDLRAD3 | 8,3056E-13 | 2,99 | 3,7736E-12 | 2,769 |
| LFNG | 6,3857E-13 | 6,062 | 2,1186E-12 | 3,433 |
| LIPG | 4,1425E-13 | 22,591 | 3,4483E-12 | 8,546 |
| LMNB1 | 4,7037E-13 | 3,373 | 9,2851E-13 | 16,211 |
| LMNB2 | 4,9044E-13 | 4,123 | 9,7276E-13 | 6,059 |
| LOX | 8,6059E-13 | 4,08 | 5,5556E-12 | 3,056 |
| LRR1 | 4,0866E-13 | 8,076 | 3,2362E-12 | 3,333 |
| LRRC17 | 7,77E-13 | 3,753 | 1,2019E-12 | 3,547 |
| LTBP1 | 4,2992E-13 | 24,089 | 4,5249E-12 | 3,774 |
| MAD2L1 | 4,7281E-13 | 9,326 | 2,0964E-12 | 7,644 |
| MASTL | 7,716E-13 | 2,842 | 2,1368E-12 | 3,478 |
| MCL1 | 4,1841E-12 | 2,69 | 1,5924E-12 | 2,95 |
| MCM10 | 3,6914E-13 | 21,604 | 1,6181E-12 | 6,295 |
| MCM2 | 4,3085E-13 | 7,686 | 1,1614E-12 | 13,054 |
| MCM3 | 4,2481E-13 | 4,489 | 1,8762E-12 | 5,174 |
| MCM5 | 4,6447E-13 | 5,404 | 1,4493E-12 | 7,333 |
| MCM7 | 5,7937E-13 | 3,554 | 1,1494E-12 | 6,458 |
| MCM8 | 4,1684E-13 | 3,832 | 1,9084E-12 | 3,464 |
| MDFIC | 5,2165E-13 | 7,158 | 2,5063E-12 | 2,748 |
| MDK | 4,2918E-13 | 5,882 | 1,0776E-12 | 6,246 |
| MELK | 3,7864E-13 | 16,754 | 1,2755E-12 | 9,115 |
| MFAP2 | 3,885E-13 | 11,296 | 1,9763E-12 | 6,53 |
| MFNG | 3,9002E-13 | 5,791 | 2,0704E-12 | 3,747 |
| MIS18A | 5,16E-13 | 2,633 | 3,8911E-12 | 3,319 |
| MIS18BP1 | 5,0125E-13 | 7,508 | 1,9157E-12 | 4,135 |
| MKI67 | 3,9277E-13 | 21,393 | 3,125E-12 | 13,1 |
| MLF1IP | 3,9355E-13 | 10,172 | 1,6639E-12 | 8,18 |
| MND1 | 4,9702E-13 | 6,352 | 2,1834E-12 | 4,259 |
| MPPED2 | 1,0309E-12 | 3,304 | 6,5789E-12 | 3,531 |
| MST4 | 6,4851E-13 | 9,814 | 4,0486E-12 | 3,609 |
| MYBL2 | 4,8567E-13 | 8,708 | 1,0582E-12 | 21,418 |
| MYT1 | 9,7276E-13 | 3,866 | 1,0684E-12 | 4,178 |
| NCAPD2 | 4,7371E-13 | 5,393 | 1,292E-12 | 7,302 |
| NCAPG | 4,0016E-13 | 10,018 | 1,0235E-12 | 6,938 |
| NCAPG2 | 9,1075E-13 | 2,618 | 1,2484E-12 | 4,019 |
| NCAPH | 3,9494E-13 | 13,447 | 1,3605E-12 | 8,989 |
| NDC80 | 3,6928E-13 | 24,782 | 1,1669E-12 | 14,762 |
| NDE1 | 3,7736E-13 | 7,911 | 7,8125E-12 | 2,596 |
| NEDD1 | 4,8239E-13 | 5,756 | 1,8349E-12 | 4,856 |
| NEIL3 | 5,4945E-13 | 6,243 | 2,3585E-12 | 3,808 |
| NEK2 | 3,9604E-13 | 17,434 | 1,2136E-12 | 7,703 |
| NES | 6,4809E-13 | 3,067 | 9,1996E-13 | 9,609 |
| NEUROD4 | 7,0077E-13 | 180,613 | 5,6497E-12 | 5,633 |
| NEUROG1 | 3,3223E-12 | 3,274 | 7,2464E-12 | 6,153 |
| **NEUROG2** | **1,7857E-11** | **6,474** | **5,5866E-12** | **14,075** |
| NHLH1 | 1,6155E-12 | 12,859 | 4,9261E-12 | 11,473 |
| **NOTCH1** | **4,0128E-13** | **8,807** | **5,618E-12** | **6,11** |
| NRARP | 2,0576E-12 | 2,655 | 1,5175E-12 | 3,283 |
| NRM | 7,3801E-13 | 3,14 | 9,5511E-13 | 4,603 |
| NUAK2 | 1,3228E-12 | 3,461 | 3,0675E-12 | 5 |
| NUF2 | 3,7313E-13 | 23,566 | 1,6129E-12 | 8,254 |
| NUP37 | 4,5372E-13 | 4,071 | 3,1153E-12 | 3,2 |
| NUSAP1 | 3,6832E-13 | 18,248 | 9,9305E-13 | 23,631 |
| NXN | 6,2228E-13 | 3,991 | 8,9686E-13 | 4,991 |
| OIP5 | 3,876E-13 | 15,038 | 1,8051E-12 | 6,99 |
| ORC1 | 6,0277E-13 | 6,46 | 2,3419E-12 | 5,728 |
| ORC6 | 6,5531E-13 | 3,481 | 3,1348E-12 | 3,699 |
| PALLD | 4,5725E-13 | 14,959 | 4,5662E-12 | 2,997 |
| PARD3 | 1,1696E-12 | 3,547 | 9,8232E-13 | 4,888 |
| PAX6 | 5,5556E-13 | 16,657 | 4,9505E-12 | 4,444 |
| PBK | 3,7965E-13 | 25,37 | 1,3123E-12 | 15,462 |
| PCNA | 4,7551E-13 | 4,694 | 2,5445E-12 | 5,893 |
| PDPN | 3,7538E-13 | 15,979 | 5,4945E-12 | 4,037 |
| PF4 | 2,1739E-11 | 2,73 | 5,3191E-12 | 2,773 |
| PGM2 | 4,9628E-13 | 3,705 | 4,2194E-12 | 2,701 |
| PHF21B | 1,0204E-12 | 3,658 | 1,0183E-12 | 6,516 |
| PHGDH | 4,6685E-13 | 7,188 | 3,7594E-12 | 3,346 |
| PHIP | 1,0267E-12 | 2,593 | 4,4444E-12 | 4,106 |
| PLAG1 | 1,2195E-12 | 3,157 | 4,1667E-12 | 3,839 |
| PLIN2 | 3,8197E-13 | 3,846 | 1,3175E-12 | 3,087 |
| PLIN3 | 4,08E-13 | 5,044 | 1,6722E-12 | 2,61 |
| PLK1 | 3,8775E-13 | 15,13 | 1,1261E-12 | 16,898 |
| PLK4 | 5,6054E-13 | 4,692 | 1,4164E-12 | 5,336 |
| POC1A | 4,5809E-13 | 7,322 | 1,9608E-12 | 4,663 |
| POLA1 | 1,4124E-12 | 3,057 | 1,9011E-12 | 3,896 |
| POLA2 | 6,5104E-13 | 4,352 | 2,3364E-12 | 3,188 |
| POLD1 | 5,1653E-13 | 3,942 | 1,4245E-12 | 6,079 |
| POLE2 | 8,658E-13 | 4,017 | 3,0303E-12 | 2,698 |
| POU3F4 | 1,3947E-12 | 3,989 | 1,5873E-12 | 7,386 |
| PPBP | 1,7544E-11 | 3,164 | 2,6954E-12 | 3,774 |
| PRC1 | 3,8285E-13 | 10,398 | 1,2563E-12 | 9,654 |
| PRIM1 | 7,0972E-13 | 2,692 | 1,0917E-12 | 3,811 |
| PRIM2 | 8,285E-13 | 3,041 | 1,2788E-12 | 3,177 |
| PRKX | 3,0303E-12 | 2,632 | 1,218E-12 | 2,764 |
| PRR11 | 4,99E-13 | 5,447 | 2,8409E-12 | 5,63 |
| PSRC1 | 4,085E-13 | 6,186 | 4,7393E-12 | 2,866 |
| PTBP1 | 4,878E-13 | 4,319 | 1,4286E-12 | 5,31 |
| PTTG1 | 3,7244E-13 | 13,002 | 1,3661E-12 | 9,997 |
| PTTG2 | 3,7258E-13 | 13,658 | 1,5504E-12 | 7,375 |
| PTTG3P | 3,7383E-13 | 12,113 | 1,3055E-12 | 11,242 |
| PTX3 | 1,0965E-12 | 2,603 | 1,3812E-12 | 3,529 |
| QSER1 | 8,1235E-13 | 2,823 | 1,4684E-12 | 3,222 |
| RACGAP1 | 4,4385E-13 | 4,556 | 1,5083E-12 | 6,018 |
| RAD51 | 3,785E-13 | 15,061 | 1,9194E-12 | 4,967 |
| RAD51AP1 | 4,4484E-13 | 13,521 | 2,0325E-12 | 5,183 |
| RAD54L | 3,7893E-13 | 20,054 | 2,2026E-12 | 3,919 |
| RAI14 | 7,4683E-13 | 2,681 | 4,1152E-12 | 3,975 |
| RBBP8 | 4,9432E-13 | 4,094 | 2,0661E-12 | 4,023 |
| RBL1 | 3,6364E-12 | 2,641 | 2,7248E-12 | 3,398 |
| RCC2 | 5,4054E-13 | 2,804 | 1,3459E-12 | 3,091 |
| RCN1 | 6,6313E-13 | 4,293 | 1,105E-12 | 3,06 |
| RDX | 7,1994E-13 | 2,593 | 1,6E-12 | 2,81 |
| REST | 7,7519E-13 | 3,642 | 3,5088E-12 | 2,603 |
| RFC3 | 1,4925E-12 | 3,409 | 1,8519E-12 | 2,804 |
| RMI2 | 4,0096E-13 | 9,947 | 1,845E-12 | 7,714 |
| RND3 | 1,7331E-12 | 2,589 | 1,1919E-12 | 5,558 |
| RNF122 | 7,0771E-13 | 2,652 | 8,5324E-13 | 4,583 |
| RPA1 | 6,689E-13 | 2,869 | 2,584E-12 | 2,715 |
| RRM2 | 3,659E-13 | 26,116 | 1,2107E-12 | 13,968 |
| RTKN2 | 5,9595E-13 | 4,509 | 1,6835E-12 | 3,887 |
| SAP30 | 3,7523E-13 | 3,922 | 1,992E-12 | 3,697 |
| SEMA5B | 3,7722E-13 | 6,885 | 2,0833E-12 | 3,544 |
| SETP10 | 4,4803E-13 | 4,498 | 3,2787E-12 | 2,773 |
| SERINC2 | 1,0753E-12 | 3,611 | 2,2676E-12 | 4,023 |
| SERTAD4 | 5,0251E-12 | 2,71 | 3,4722E-12 | 3,592 |
| SFRP2 | 4,529E-13 | 14,669 | 1,3947E-12 | 9,704 |
| SGOL1 | 3,7779E-13 | 18,902 | 1,7889E-12 | 5,288 |
| SGOL2 | 4,902E-13 | 6,23 | 1,7544E-12 | 4,851 |
| SHCBP1 | 4,0502E-13 | 13,628 | 2,0576E-12 | 4,982 |
| SHMT2 | 4,6125E-13 | 3,036 | 2,9326E-12 | 2,847 |
| SHROOM3 | 6,7204E-13 | 3,753 | 6,993E-12 | 2,94 |
| SKA1 | 3,8745E-13 | 13,466 | 2,2422E-12 | 4,611 |
| SKA3 | 3,7481E-13 | 15,163 | 1,4327E-12 | 6,376 |
| SKP2 | 1,261E-12 | 3,086 | 1,4006E-12 | 5,501 |
| SLC1A5 | 5,7307E-13 | 5,109 | 1,2453E-12 | 4,713 |
| SMAD5 | 1,199E-12 | 3,203 | 2,439E-12 | 2,907 |
| SMC1A | 4,3821E-13 | 3,361 | 9,7466E-13 | 3,027 |
| SMC2 | 5,4113E-13 | 4,966 | 1,6863E-12 | 4,517 |
| SMC4 | 4,0683E-13 | 7,593 | 1,3624E-12 | 11,474 |
| SMC5 | 3,3557E-12 | 2,734 | 1,6667E-12 | 2,593 |
| SMO | 3,7023E-13 | 8,225 | 1,368E-12 | 4,5 |
| SNCAIP | 4,1859E-13 | 3,12 | 1,1249E-12 | 3,502 |
| SOX1 | 4,0388E-13 | 11,416 | 8,5616E-13 | 11,79 |
| SOX11 | 9,0253E-13 | 2,969 | 8,5616E-13 | 11,79 |
| SOX2 | 3,87E-13 | 12,151 | 1,0741E-12 | 8,911 |
| **SOX3** | **4,1237E-13** | **40,29** | **3,367E-12** | **18,537** |
| SOX9 | 4,5914E-13 | 6,323 | 5,9172E-12 | 5,644 |
| SP1 | 4,6168E-13 | 5,513 | 3,2258E-12 | 3,214 |
| SPAG5 | 3,8956E-13 | 11,098 | 1,7271E-12 | 7,341 |
| SPATA13 | 5,6338E-13 | 3,07 | 3,8168E-12 | 3,075 |
| SPC24 | 3,9841E-13 | 12,236 | 1,1481E-12 | 11,964 |
| SPC25 | 3,7189E-13 | 21,145 | 1,3477E-12 | 10,244 |
| STAG1 | 8,1766E-13 | 3,112 | 8,9206E-13 | 2,875 |
| STIL | 5,5741E-13 | 5,328 | 3,4843E-12 | 2,907 |
| STON1 | 3,7286E-13 | 10,158 | 2,8986E-12 | 3,845 |
| STON1-GTF2A1L | 1,9493E-12 | 5,122 | 4,7619E-12 | 3,664 |
| SVIL | 7,4906E-13 | 3,24 | 6,4103E-12 | 2,744 |
| TACC3 | 4,0933E-13 | 12,306 | 1,2642E-12 | 9,042 |
| TCF19 | 7,5815E-13 | 3,127 | 1,227E-12 | 4,385 |
| TCF3 | 3,9324E-13 | 4,045 | 9,9108E-13 | 7,157 |
| TCF7L1 | 2,3923E-12 | 3,785 | 6,2893E-12 | 4,288 |
| TEAD1 | 5,4975E-13 | 3,126 | 1,1737E-12 | 3,391 |
| **TEAD2** | **4,4623E-13** | **5,235** | **1,0537E-12** | **10,225** |
| TFDP2 | 4,8356E-13 | 3,301 | 4,878E-12 | 2,706 |
| **TGIF2** | **3,647E-13** | **12,003** | **1,1891E-12** | **6,991** |
| TIFA | 1,0504E-12 | 2,755 | 6,1728E-12 | 3,893 |
| TIMELESS | 4,3403E-13 | 5,411 | 1,1236E-12 | 9,478 |
| TK1 | 4,1169E-13 | 9,481 | 1,8868E-12 | 6,463 |
| TMEM123 | 4,8054E-13 | 6,946 | 7,1942E-12 | 4,054 |
| TMEM194A | 4,931E-13 | 5,337 | 3,3333E-12 | 3,402 |
| TMEM98 | 3,712E-13 | 6,262 | 2,681E-12 | 3,548 |
| TMPO | 4,3783E-13 | 5,564 | 1,0101E-12 | 7,478 |
| TMX1 | 4,6773E-13 | 4,1 | 5,8824E-12 | 2,999 |
| TNFRSF19 | 2,9326E-12 | 2,765 | 3,096E-12 | 4,458 |
| TONSL | 2,1231E-12 | 2,795 | 2,1978E-12 | 2,743 |
| TOP2A | 3,6969E-13 | 33,203 | 9,6061E-13 | 40,183 |
| TP53 | 4,5579E-13 | 7,022 | 1,9455E-12 | 5,083 |
| TPX2 | 4,3516E-13 | 9,788 | 1,0965E-12 | 18,479 |
| TRIM59 | 3,7202E-13 | 10,847 | 2,1053E-12 | 6,471 |
| TRIP10 | 9,3284E-13 | 3,436 | 2,3474E-12 | 2,738 |
| TRIP13 | 1,3643E-12 | 3,921 | 1,773E-12 | 3,684 |
| TROAP | 3,7106E-13 | 17,635 | 2,8818E-12 | 5,325 |
| TSPAN6 | 4,5167E-13 | 3,834 | 1,2225E-12 | 3,816 |
| TTK | 4,2159E-13 | 15,243 | 1,2953E-12 | 8,516 |
| TULP3 | 9,3897E-13 | 2,963 | 2,2222E-12 | 2,712 |
| TYMS | 3,8417E-13 | 10,011 | 1,1038E-12 | 15,044 |
| UBE2C | 3,6805E-13 | 21,934 | 9,99E-13 | 25,045 |
| UHRF1 | 3,8685E-13 | 12,208 | 1,182E-12 | 11,138 |
| VCAM1 | 5,7045E-13 | 9,795 | 1,6234E-12 | 3,996 |
| VEPH1 | 1,4793E-12 | 3,841 | 1,7921E-12 | 4,957 |
| VIM | 4,3802E-13 | 5,274 | 9,7182E-13 | 9,663 |
| WDHD1 | 4,7348E-13 | 4,388 | 1,8182E-12 | 4,219 |
| WDR62 | 1,0395E-12 | 2,941 | 3,1447E-12 | 3,471 |
| WDR76 | 6,5402E-13 | 3,992 | 1,7007E-12 | 4,996 |
| WEE1 | 3,9746E-13 | 11,268 | 1,2315E-12 | 6,037 |
| WSCD1 | 1,1779E-12 | 2,959 | 1,0695E-12 | 3,039 |
| YAP1 | 5,4855E-13 | 8,917 | 1,5798E-12 | 5,144 |
| ZFHX4 | 5,7571E-13 | 9,426 | 4,7847E-12 | 3,235 |
| ZFP36L1 | 5,5463E-13 | 4,769 | 4,4843E-12 | 5,534 |
| ZFP36L2 | 5,9916E-13 | 4,659 | 4,6083E-12 | 3,745 |
| ZIK1 | 4,5977E-13 | 2,668 | 2,1786E-12 | 2,587 |
| ZNF217 | 6,1501E-13 | 4,648 | 6,6667E-12 | 3,733 |
| ZNF367 | 4,6729E-13 | 8,963 | 3,7313E-12 | 3,906 |
| ZNF516 | 1,8622E-12 | 3,169 | 1,5974E-12 | 3,163 |
| ZNF93 | 5,152E-13 | 2,627 | 1,0753E-12 | 3,08 |
| ZWINT | 4,0306E-13 | 9,99 | 1,269E-12 | 9,222 |
