# Supplementary material for: A multi-resource data integration approach: identification of candidate genes regulating cell proliferation during neocortical development
Source: Front Neurosci. 2014 Aug 21;8:257. doi: 10.3389/fnins.2014.00257 (PMC4139594; doi:10.3389/fnins.2014.00257)
Supplement: Supplementary file 1 [file DataSheet1.ZIP › 108271_Nowakowski_Table_6.DOCX]

**Table S6A. Combination of key words (Key Word 1+Key Word 2) searched by PubMed and the numbers of articles retrieved (A)**. Only the 13 mouse VZ candidate genes are included. “*” indicates numbers that were controlled by advanced search “[Title/Abstract]”.

| Key Word 1  Key Word 2 | Cdon(Cdo) | Celsr1 | Dbi | E2f5 | Eomes | Hmgn2 | Neurog2 | Notch1 | Pcnt | Sox3 | Ssrp1 | Tead2 | Tgif2 |
| --- | --- | --- | --- | --- | --- | --- | --- | --- | --- | --- | --- | --- | --- |
| Cdon(Cdo) | 42(450) |  |  |  |  |  |  |  |  |  |  |  |  |
| Celsr1 | 0(0) | 63 |  |  |  |  |  |  |  |  |  |  |  |
| Dbi | 0(0) | 0 | 679 |  |  |  |  |  |  |  |  |  |  |
| E2f5 | 0(0) | 0 | 0 | 144 |  |  |  |  |  |  |  |  |  |
| Eomes | 0(0) | 0 | 0 | 0 | 301 |  |  |  |  |  |  |  |  |
| Hmgn2 | 0(0) | 0 | 0 | 0 | 0 | 263 |  |  |  |  |  |  |  |
| Neurog2 | 0(0) | 0 | 0 | 0 | 5 | 0 | 199 |  |  |  |  |  |  |
| Notch1 | 0(0) | 0 | 1 | 0 | 1 | 0 | 4 | *2528 |  |  |  |  |  |
| Pcnt | 0(0) | 0 | 0 | 0 | 0 | 0 | 0 | 0 | 48 |  |  |  |  |
| Sox3 | 0(0) | 0 | 0 | 0 | 1 | 0 | 1 | 1 | 0 | 291 |  |  |  |
| Ssrp1 | 0(0) | 0 | 0 | 0 | 0 | 0 | 0 | 0 | 0 | 0 | 109 |  |  |
| Tead2 | 0(0) | 0 | 0 | 0 | 1 | 1 | 0 | 1 | 0 | 0 | 0 | 37 |  |
| Tgif2 | 0(0) | 0 | 0 | 0 | 0 | 0 | 0 | 0 | 0 | 0 | 0 | 0 | 26 |

**Table S6B. Combination of key words (Key Word 1+Key Word 2) searched by PubMed and the numbers of articles retrieved (B).** 28 genes (obtained by the searching strategy in **Table S6A**) that interact with the 13 candidates are included. “*” indicates numbers that were controlled by advanced search “[Title/Abstract]”.

| Key Word 1  Key Word 2 | Akt | Ctnnb1 | Cav1 | Dbx1 | Dll1 | Dll4 | Dtx1 | Egfr | Foxn4 | Gli3 | Gsk3 | Hes1 | P53 | Pax3 |
| --- | --- | --- | --- | --- | --- | --- | --- | --- | --- | --- | --- | --- | --- | --- |
| Akt |  |  |  |  |  |  |  |  |  |  |  |  |  |  |
| Ctnnb1 | 1115 |  |  |  |  |  |  |  |  |  |  |  |  |  |
| Cav1 | 58 | 21 |  |  |  |  |  |  |  |  |  |  |  |  |
| Dbx1 | 0 | 0 | 0 |  |  |  |  |  |  |  |  |  |  |  |
| Dll1 | 1 | 5 | 0 | 0 |  |  |  |  |  |  |  |  |  |  |
| Dll4 | 14 | 9 | 0 | 0 | 61 |  |  |  |  |  |  |  |  |  |
| Dtx1 | 0 | 0 | 0 | 0 | 1 | 0 |  |  |  |  |  |  |  |  |
| Egfr | *2242 | 325 | 37 | 0 | 2 | 5 | 0 |  |  |  |  |  |  |  |
| Foxn4 | 0 | 0 | 0 | 0 | 0 | 4 | 0 | 0 |  |  |  |  |  |  |
| Gli3 | 7 | 18 | 0 | 1 | 0 | 0 | 0 | 4 | 0 |  |  |  |  |  |
| Gsk3 | *354 | *1 | 6 | 0 | 0 | 1 | 0 | 51 | 0 | 8 |  |  |  |  |
| Hes1 | 38 | 36 | 1 | 1 | 44 | 29 | 7 | 9 | 0 | 4 | 14 |  |  |  |
| P53 | *1671 | 1007 | 38 | 0 | 4 | 8 | 3 | 1370 | 0 | 5 | 159 | 26 |  |  |
| Pax3 | 14 | 27 | 1 | 3 | 4 | 1 | 0 | 2 | 0 | 5 | 5 | 7 | 38 |  |
| Pax6 | 10 | 34 | 2 | 9 | 1 | 2 | 0 | 14 | 2 | 15 | 4 | 19 | 17 | 49 |
| Pou4f2 | 0 | 1 | 0 | 0 | 0 | 0 | 0 | 0 | 0 | 0 | 0 | 1 | 1 | 0 |
| Prox1 | 11 | 7 | 0 | 0 | 0 | 4 | 0 | 0 | 4 | 0 | 0 | 1 | 6 | 0 |
| Ptf1a | 1 | 5 | 0 | 1 | 1 | 0 | 0 | 1 | 4 | 0 | 0 | 5 | 5 | 0 |
| Rbpj | 7 | 10 | 0 | 0 | 8 | 4 | 3 | 0 | 0 | 1 | 5 | 50 | 8 | 1 |
| Rnd2 | 0 | 0 | 0 | 0 | 0 | 0 | 0 | 0 | 0 | 0 | 0 | 0 | 1 | 0 |
| Shh | 79 | 172 | 1 | 6 | 1 | 1 | 0 | 32 | 2 | 289 | 19 | 23 | 54 | 25 |
| Smad2 | 170 | 86 | 7 | 0 | 1 | 1 | 0 | 20 | 0 | 1 | 18 | 3 | 57 | 1 |
| Sox2 | 68 | 122 | 1 | 1 | 0 | 0 | 2 | 32 | 2 | 7 | 34 | 28 | 112 | 7 |
| Sox9 | 21 | 118 | 0 | 0 | 0 | 0 | 0 | 9 | 0 | 10 | 7 | 21 | 22 | 20 |
| Tgif | 6 | 1 | 0 | 0 | 0 | 0 | 0 | 4 | 0 | 3 | 0 | 0 | 0 | 0 |
| Tlx | 1 | 1 | 0 | 0 | 0 | 0 | 0 | 0 | 0 | 2 | 1 | 2 | 4 | 0 |
| Wnt7a | 5 | 82 | 0 | 0 | 0 | 0 | 0 | 3 | 0 | 4 | 9 | 0 | 2 | 0 |
| Yap | 43 | 48 | 1 | 0 | 0 | 0 | 0 | 29 | 0 | 1 | 7 | 0 | 64 | 2 |

(Table S6B continued)

| Key Word 1  Key Word 2 | Pax6 | Pou4f2 | Prox1 | Ptf1a | Rbpj | Rnd2 | Shh | Smad2 | Sox2 | Sox9 | Tgif | Tlx | Wnt7a | Yap |
| --- | --- | --- | --- | --- | --- | --- | --- | --- | --- | --- | --- | --- | --- | --- |
| Pou4f2 | 6 |  |  |  |  |  |  |  |  |  |  |  |  |  |
| Prox1 | 42 | 1 |  |  |  |  |  |  |  |  |  |  |  |  |
| Ptf1a | 5 | 1 | 3 |  |  |  |  |  |  |  |  |  |  |  |
| Rbpj | 2 | 1 | 0 | 15 |  |  |  |  |  |  |  |  |  |  |
| Rnd2 | 0 | 0 | 0 | 0 | 0 |  |  |  |  |  |  |  |  |  |
| Shh | 84 | 1 | 2 | 4 | 4 | 0 |  |  |  |  |  |  |  |  |
| Smad2 | 1 | 0 | 0 | 0 | 0 | 0 | 6 |  |  |  |  |  |  |  |
| Sox2 | 173 | 1 | 20 | 2 | 4 | 0 | 65 | 12 |  |  |  |  |  |  |
| Sox9 | 14 | 0 | 3 | 8 | 6 | 0 | 42 | 17 | 76 |  |  |  |  |  |
| Tgif | 1 | 0 | 0 | 0 | 0 | 0 | 45 | 27 | 1 | 1 |  |  |  |  |
| Tlx | 4 | 0 | 0 | 0 | 1 | 0 | 3 | 1 | 6 | 0 | 0 |  |  |  |
| Wnt7a | 1 | 0 | 0 | 0 | 0 | 0 | 31 | 1 | 0 | 0 | 6 | 1 |  |  |
| Yap | 5 | 0 | 0 | 0 | 1 | 0 | 7 | 3 | 8 | 5 | 0 | 1 | 0 |  |
